# Evaluation of an Educational Outreach and Audit and Feedback Program to Reduce Continuous Pulse Oximetry Use in Hospitalized Infants With Stable Bronchiolitis: A Nonrandomized Clinical Trial

**DOI:** 10.1001/jamanetworkopen.2021.22826

**Published:** 2021-09-02

**Authors:** Amanda C. Schondelmeyer, Amanda P. Bettencourt, Rui Xiao, Rinad S. Beidas, Courtney Benjamin Wolk, Christopher P. Landrigan, Patrick W. Brady, Canita R. Brent, Padmavathy Parthasarathy, Andrew S. Kern-Goldberger, Nathaniel Sergay, Vivian Lee, Christopher J. Russell, Julianne Prasto, Sarah Zaman, Kaitlyn McQuistion, Kate Lucey, Courtney Solomon, Mayra Garcia, Christopher P. Bonafide

**Affiliations:** 1 Department of Systems, Populations, and Leadership, University of Michigan School of Nursing, Ann Arbor; 2 Department of Biostatistics, Epidemiology, and Informatics, Perelman School of Medicine at the University of Pennsylvania, Philadelphia; 3 Department of Psychiatry, Perelman School of Medicine at the University of Pennsylvania, Philadelphia; 4 Department of Medical Ethics and Health Policy, Perelman School of Medicine at the University of Pennsylvania, Philadelphia; 5 Department of Medicine, Perelman School of Medicine at the University of Pennsylvania, Philadelphia; 6 Penn Implementation Science Center at the Leonard Davis Institute of Health Economics (PISCE@LDI), University of Pennsylvania, Philadelphia; 7 Division of General Pediatrics, Department of Pediatrics, Boston Children’s Hospital, Boston, Massachusetts; 8 Division of Sleep and Circadian Disorders, Departments of Medicine, Brigham and Women’s Hospital, Boston, Massachusetts; 9 Department of Pediatrics, Harvard Medical School, Boston, Massachusetts; 10 Division of Sleep and Circadian Disorders, Department of Neurology, Brigham and Women’s Hospital, Boston, Massachusetts; 11 Department of Pediatrics, University of Cincinnati College of Medicine, Cincinnati, Ohio; 12 Division of Hospital Medicine, Cincinnati Children’s Hospital Medical Center, Cincinnati, Ohio; 13 James M. Anderson Center for Health Systems Excellence, Cincinnati Children’s Hospital Medical Center, Cincinnati, Ohio; 14 Section of Pediatric Hospital Medicine, Children’s Hospital of Philadelphia, Philadelphia, Pennsylvania; 15 Center for Pediatric Clinical Effectiveness, Children’s Hospital of Philadelphia, Philadelphia, Pennsylvania; 16 Department of Pediatrics, Perelman School of Medicine at the University of Pennsylvania, Philadelphia; 17 Pediatric Residency Program, Children’s Hospital of Philadelphia, Philadelphia, Pennsylvania; 18 Division of Hospital Medicine, Children’s Hospital Los Angeles, Los Angeles, California; 19 Department of Pediatrics, Keck School of Medicine, University of Southern California, Los Angeles; 20 Division of Pediatrics, Children’s Hospital of Philadelphia Pediatric Care and Penn Medicine Princeton Medical Center, Philadelphia, Pennsylvania; 21 Department of Pediatrics, University of Washington School of Medicine, Seattle; 22 Center for Child Health, Behavior, and Development, Seattle Children’s Research Institute, Seattle, Washington; 23 University of Washington Pediatric Residency Program, Department of Pediatrics, University of Washington, Seattle; 24 Division of Hospital Medicine, Ann & Robert H. Lurie Children’s Hospital of Chicago, Chicago, Illinois; 25 Department of Pediatrics, Northwestern University Feinberg School of Medicine, Chicago, Illinois; 26 Department of Pediatrics, UT Southwestern Medical Center, Dallas, Texas; 27 Division of Pediatric Hospital Medicine, Children’s Health Dallas, Texas; 28 Division of General and Thoracic Surgery, Children’s Health Dallas, Dallas, Texas

## Abstract

**Importance:**

National guidelines recommend against continuous pulse oximetry use for hospitalized children with bronchiolitis who are not receiving supplemental oxygen, yet guideline-discordant use remains high.

**Objectives:**

To evaluate deimplementation outcomes of educational outreach and audit and feedback strategies aiming to reduce guideline-discordant continuous pulse oximetry use in children hospitalized with bronchiolitis who are not receiving supplemental oxygen.

**Design, Setting, and Participants:**

A nonrandomized clinical single-group deimplementation trial was conducted in 14 non–intensive care units in 5 freestanding children’s hospitals and 1 community hospital from December 1, 2019, through March 14, 2020, among 847 nurses and physicians caring for hospitalized children with bronchiolitis who were not receiving supplemental oxygen.

**Interventions:**

Educational outreach focused on communicating details of the existing guidelines and evidence. Audit and feedback strategies included 2 formats: (1) weekly aggregate data feedback to multidisciplinary teams with review of unit-level and hospital-level use of continuous pulse oximetry, and (2) real-time 1:1 feedback to clinicians when guideline-discordant continuous pulse oximetry use was discovered during in-person data audits.

**Main Outcomes and Measures:**

Clinician ratings of acceptability, appropriateness, feasibility, and perceived safety were assessed using a questionnaire. Guideline-discordant continuous pulse oximetry use in hospitalized children was measured using direct observation of a convenience sample of patients with bronchiolitis who were not receiving supplemental oxygen.

**Results:**

A total of 847 of 1193 eligible clinicians (695 women [82.1%]) responded to a Likert scale-based questionnaire (71% response rate). Most respondents rated the deimplementation strategies of education and audit and feedback as acceptable (education, 435 of 474 [92%]; audit and feedback, 615 of 664 [93%]), appropriate (education, 457 of 474 [96%]; audit and feedback, 622 of 664 [94%]), feasible (education, 424 of 474 [89%]; audit and feedback, 557 of 664 [84%]), and safe (803 of 847 [95%]). Sites collected 1051 audit observations (range, 47-403 per site) on 709 unique patient admissions (range, 31-251 per site) during a 3.5-month period of continuous pulse oximetry use in children with bronchiolitis not receiving supplemental oxygen, which were compared with 579 observations (range, 57-154 per site) from the same hospitals during the baseline 4-month period (prior season) to determine whether the strategies were associated with a reduction in use. Sites conducted 148 in-person educational outreach and aggregate data feedback sessions and provided real-time 1:1 feedback 171 of 236 times (72% of the time when guideline-discordant monitoring was identified). Adjusted for age, gestational age, time since weaning from supplemental oxygen, and other characteristics, guideline-discordant continuous pulse oximetry use decreased from 53% (95% CI, 49%-57%) to 23% (95% CI, 20%-25%) (*P* < .001) during the intervention period. There were no adverse events attributable to reduced monitoring.

**Conclusions and Relevance:**

In this nonrandomized clinical trial, educational outreach and audit and feedback deimplementation strategies for guideline-discordant continuous pulse oximetry use among hospitalized children with bronchiolitis who were not receiving supplemental oxygen were positively associated with clinician perceptions of feasibility, acceptability, appropriateness, and safety. Evaluating the sustainability of deimplementation beyond the intervention period is an essential next step.

**Trial Registration:**

ClinicalTrials.gov Identifier: NCT04178941

## Key Points

**Question:**

Are audit and feedback strategies and educational outreach associated with clinician perceptions of the feasibility, acceptability, appropriateness, and safety of continuous pulse oximetry use in children hospitalized with bronchiolitis who are not receiving supplemental oxygen (guideline-discordant use)?

**Findings:**

In this 6-hospital single-group nonrandomized clinical trial, 847 nurses and physicians highly rated the feasibility, acceptability, and appropriateness of audit and feedback strategies and educational outreach. Guideline-discordant pulse oximetry use decreased from 53% to 23% during the intervention period.

**Meaning:**

Educational outreach and audit and feedback strategies were feasible, acceptable, appropriate, and associated with a reduction in guideline-discordant continuous pulse oximetry use in children hospitalized with bronchiolitis who are not receiving supplemental oxygen.

## Teaser

This nonrandomized clinical trial evaluates deimplementation outcomes of educational outreach and audit and feedback strategies aiming to reduce guideline-discordant continuous pulse oximetry use in children hospitalized with bronchiolitis who are not receiving supplemental oxygen.

## Introduction

Bronchiolitis is the leading reason for non–birth-related hospitalization in infants, accounting for more than 100 000 hospitalizations and $734 million in hospital costs annually.^1^ Bronchiolitis is a self-limited viral illness with a well-documented clinical course and national evidence-based practice guidelines.^2,3,4^ In addition to management recommendations, guidelines advise against continuous pulse oximetry monitoring (cSpo_2_) for patients who are not receiving supplemental oxygen, as it increases health care use without improving outcomes.^2,3,5,6,7,8,9^ Although none of the guidelines that discourage cSpo_2_ explicitly define it, we consider any use of pulse oximetry beyond a spot check (an in-person assessment of the oxygen saturation, with the probe applied and removed by staff during a single visit to the patient’s room) to represent continuous measurement. A recent study measured cSpo_2_ use at 56 US and Canadian hospitals and found that, overall, 46% of patients received cSpo_2_, suggesting a gap between the evidence-based guidelines and real-world clinical practice.^10^

Implementation science seeks to overcome evidence-to-practice gaps.^11,12^ Audit and feedback and educational outreach are common implementation strategies that are effective in improving processes of care and clinical outcomes^13^ and are used frequently and successfully in quality improvement initiatives in pediatric hospital settings.^14,15,16,17^ However, the feasibility of audit and feedback and educational outreach and their associations with successful deimplementation in the pediatric hospital setting is limited mainly to quality improvement collaboratives, with diverse bundled interventions and multiple practices targeted for deimplementation.^18,19^

In this study, our objective was to measure the feasibility, acceptability, appropriateness, and perceived safety of educational outreach and audit and feedback deimplementation strategies and their effect on cSpo_2_ use in hospitalized children with bronchiolitis who are not receiving supplemental oxygen.

## Methods

This nonrandomized clinical trial was part of the Eliminating Monitor Overuse: SpO_2_ portfolio of projects, which previously measured baseline cSpo_2_ use in patients with bronchiolitis who were not receiving supplemental oxygen from December 1, 2018, through March 31, 2019, at 56 hospitals (trial protocol in Supplement 1).^10^ The intervention took place in 14 non–intensive care units in 5 freestanding children’s hospitals and 1 community hospital from December 1, 2019, through March 14, 2020. The institutional review board at Children’s Hospital of Philadelphia approved the study, and the remaining sites established reliance agreements with the reviewing institutional review board. All sites granted waivers of consent or parental permission per 45 CFR 46.116(f)(3), assent per 45 CFR 46.408(a), and Health Insurance Portability and Accountability Act authorization per 45 CFR 164.512(i)(2)(ii). For the staff questionnaires, sites granted waivers of consent documentation per 45 CFR 46.117(c)(1)(ii). This study followed the Transparent Reporting of Evaluations With Nonrandomized Designs (TREND) reporting guideline.

### Design

This was a prospective, nonrandomized, single-group implementation feasibility trial with historical control data from the baseline period listed above. We invited hospitals that participated in the baseline study to express interest in pilot trial participation using an online form. Twenty-three hospitals expressed interest. In addition to the primary study site (Children’s Hospital of Philadelphia), we invited 5 sites with risk-standardized monitoring percentages of 60% or more during the baseline study aiming to optimize diversity of geography and community hospital participation, as well as availability of monitor data in the electronic health record for a related substudy.^20^

**Figure 1.**
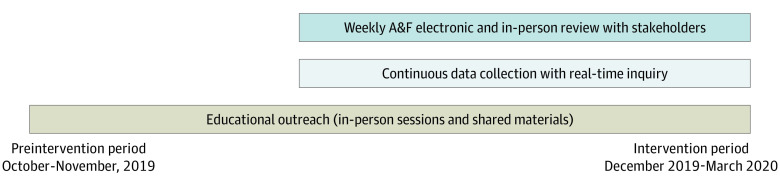
Study Timeline Timeline depicting the intervention including data collection, audit and feedback (A&F), and educational outreach interventions. Audit and feedback intervention consisted of bedside data collection on continuous pulse oximetry monitoring status (the audit) and 2 forms of feedback: individual real-time inquiry conducted at the time of data collection and weekly unit-level performance feedback. Educational outreach intervention consisted of the 3 core components, which included national guidelines for pulse oximetry monitoring in patients with bronchiolitis, evidence supporting intermittent rather than continuous pulse oximetry monitoring, and the hospital’s baseline and current pulse oximetry monitoring performance.

The intervention consisted of 2 deimplementation strategies: educational outreach and audit and feedback (Figure 1). These strategies were chosen based on barriers and facilitators identified in prior qualitative research^21^ and were then mapped to deimplementation strategies during 2 stakeholder strategy development panels. The education and audit and feedback interventions were delivered in person to physicians, nurses, and respiratory therapists.

### Educational Outreach Intervention

Educational outreach started before audit and feedback, then continued at a frequency determined by the site principal investigator based on study site needs. Education included 3 components: national guidelines for cSpo_2_ monitoring in patients with bronchiolitis,^2,3,4^ evidence supporting intermittent pulse oximetry rather than cSpo_2_ monitoring, and their hospital’s baseline cSpo_2_ use^10^ (eFigure 1 in Supplement 2). Site principal investigators tailored non–core content (eg, logos and location-specific context) and the setting of sessions. Sites were asked to target a 50% reduction in cSpo_2_ use.

### Audit and Feedback Intervention and cSpo_2_ Use Data Collection

Study team members underwent webinar-based training in the fall of 2019. During the intervention period, local study teams conducted medical record review to screen patients for eligibility, followed by bedside data collection rounds using previously published methods^10,22^ to determine the cSpo_2_ status of patients with bronchiolitis not receiving supplemental oxygen. These data served as the outcome measure for cSpo_2_ use and the audit data for audit and feedback. Teams were encouraged to conduct data collection twice weekly, with timing based on availability of the data collector. Given that rapid improvements in clinical status are expected with bronchiolitis^23^ and that cSpo_2_ use should change accordingly, sites could collect data from the same patient on multiple occasions if observations were more than 6 hours apart. An identifier allowed accounting for clustering in analyses. Eligible patients were between the ages of 8 weeks and 23 months and admitted to a general medical service with a primary diagnosis of bronchiolitis. We excluded patients documented as premature or preterm and those with documented prematurity of less than 28 weeks’ gestation; cyanotic congenital heart disease or pulmonary hypertension; home oxygen use, positive pressure ventilation requirement, or tracheostomy; primary neuromuscular disease; immunodeficiency; or cancer. Additional exclusions were added in December 2019 (heart failure, myocarditis, or arrhythmia) and in March 2020 (COVID-19).

The feedback intervention had 2 forms: individual real-time inquiry and weekly unit-level feedback. In real-time inquiry, if clinicians were available during cSpo_2_ audits, data collectors asked briefly and nonjudgmentally about the indication for cSpo_2_. In unit-level feedback, the study coordinating team summarized each participating unit’s data in a weekly unit-specific dashboard (an electronic document with unit-specific cSpo_2_ monitoring performance and reminders for specific practices to improve performance) sent to site principal investigators that reiterated educational outreach information (eFigure 2 in Supplement 2). In the dashboard, which site principal investigators tailored to site needs, data were compared with the performance of other hospital units, the hospital’s baseline performance,^10^ and the hospital’s target performance. The study coordinating team also suggested improvement targets based on local monitoring patterns, such as day and night variation.

### Outcomes

The primary outcomes were acceptability, appropriateness, and feasibility of the deimplementation strategies.^21^ We also measured perceived safety of intermittently spot-checking oxygen saturation instead of using cSpo_2_. To estimate penetration^24^ of guideline-concordant care^2,3,4^ of patients with bronchiolitis not receiving supplemental oxygen, we assessed the change in cSpo_2_ use between the baseline^10^ and intervention periods.

#### Implementation Outcomes

Aiming to distribute a brief instrument with minimal overlap between questions, our multidisciplinary study team of experts in pediatrics, nursing, clinical research, patient safety, and implementation science parsimoniously selected and adapted items from the validated Acceptability of Intervention Measure, Intervention Appropriateness Measure, and Feasibility of Intervention Measure instruments^25^ for the study questionnaire. The team also developed questionnaire items focused on perceived safety, norms, and intentions using published guidance on constructing Theory of Planned Behavior–based questionnaires.^26^ The resulting questionnaire (eAppendix in Supplement 2) was distributed electronically to study unit clinicians, including nurses, advanced practice nurses, and resident, fellow, and attending physicians. Respiratory therapists were not included because their scope of practice does not include managing cSpo_2_ monitoring and their coverage is spread across multiple hospital units, making it difficult to identify individuals who worked on intervention units. Clinicians who reported caring for patients with bronchiolitis on intervention units but not being exposed to either intervention only completed the questions about perceived safety, norms, and intentions.

#### Clinical Outcomes

We report the percentage of patients with bronchiolitis not receiving supplemental oxygen who were receiving cSpo_2_ as “guideline-discordant monitoring” in the baseline and intervention periods. To report the estimated change in penetration^24,27^ of guideline-concordant care (avoiding cSpo_2_ for patients not receiving supplemental oxygen), we examined the reduction in cSpo_2_ between the baseline and intervention periods.

Patient characteristics were abstracted from the electronic health record, including age, history of prematurity, sex, race, ethnicity, time since weaning from supplemental oxygen, history of apnea or a condition associated with neurologic impairment (eg, cerebral palsy), and presence of an enteral feeding tube.

### Adverse Event Surveillance

We performed active surveillance for adverse events that could be associated with reductions in cSpo_2_. Staff at each site screened locally available data for code blue and rapid response team activations in any patients with bronchiolitis hospitalized on study units. Medical records of patients meeting these criteria were reviewed, and staff involved in the event were interviewed if necessary. If the patient was unmonitored during the event, there was further investigation. Events were considered at least possibly related to the study intervention if there was “a reasonable possibility that the adverse event may have been caused by the procedures involved in the research.”^28^^(p1)^ Events determined to be at least possibly related to the study intervention were escalated according to local institutional review board protocols.

### Statistical Analysis

For the questionnaire-based outcomes, we first summarized responses to each question descriptively. We then explored differences in responses between nurses and physicians using Pearson χ^2^ tests. We used ordinal logistic regression accounting for hospital-level clustering and reported odds ratios (ORs) with 95% CIs for nurses, with physicians as the reference group. The OR in ordinal logistic regression indicates the odds of choosing a response on the Likert scale 1 unit higher in agreement vs a response less than or equal to that level.^29^ Because these ORs can be difficult to interpret, for questions with significant differences in agreement between nurses and physicians, we calculated predictive marginal probabilities of each level of agreement^29^ and compared them by profession. We did not make any adjustments for multiple comparisons.^30^

We calculated unadjusted guideline-discordant monitoring percentages for each hospital using the denominator of all directly observed patients with bronchiolitis who were not receiving supplemental oxygen and the numerator of patients who were simultaneously receiving cSpo_2_. We further compared baseline and intervention period data overall and at the hospital level using logistic regression, adjusted for the same covariates used in previous research, including age combined with preterm birth, time since weaning from supplemental oxygen, documented history of apnea or cyanosis during the present illness, presence of an enteral feeding tube, neurological impairment, and nighttime observation.^10^ To obtain adjusted estimates at the hospital level, we included an interaction term between hospital and the intervention period. The model accounted for clustering of observations within patient admissions.

To report the estimated change in penetration^24,27^ of guideline-concordant care (avoiding cSpo_2_ for patients with bronchiolitis who were not receiving supplemental oxygen), we calculated the difference in the cSpo_2_ use percentage between the baseline and intervention periods, with the percentage point decrease in guideline-discordant monitoring corresponding to an equivalent percentage point increase in the penetration of guideline-concordant care.

Prior to the study, we performed a power calculation based on the 5-point Likert-based Acceptability of Intervention Measure, Intervention Appropriateness Measure, and Feasibility of Intervention Measure questionnaire. Assuming an average feasibility response of “agree” (numerical value of 4.0) with an SD of 3 and alpha = 0.05, a sample size of 73 questionnaires per site would provide 80% power to reject the null hypothesis of a “neither agree nor disagree” response (numerical value of 3.0) at each site. We used data collection forms designed in Research Electronic Data Capture and hosted at Children’s Hospital of Philadelphia.^31^ We used Stata, version 16.0 (StataCorp LLC) for all analyses. All *P* values were from 2-sided tests and results were deemed statistically significant at *P* < .05.

## Results

The intervention period took place in 14 units inside 6 hospitals and included 1051 audit observations (range, 47-403 per site) on 709 unique patient admissions (range, 31-251 per site) between December 1, 2019, and March 14, 2020. The initially planned study period extended to March 31, 2020, but closed early because of institutional restrictions imposed during the COVID-19 pandemic. During the intervention period, sites completed a median of 16 in-person education and/or data feedback sessions (range, 10-64 per site). Audit observations during the study period were compared with 579 observations (range, 57-154 per site) from the same hospitals during the baseline 4-month period (prior season) to determine if the strategies were associated with a reduction in use. Sites conducted 148 in-person educational outreach and aggregate data feedback sessions and provided real-time 1:1 feedback 171 of 236 times (72% of the time when guideline-discordant monitoring was identified).

**Figure 2.**
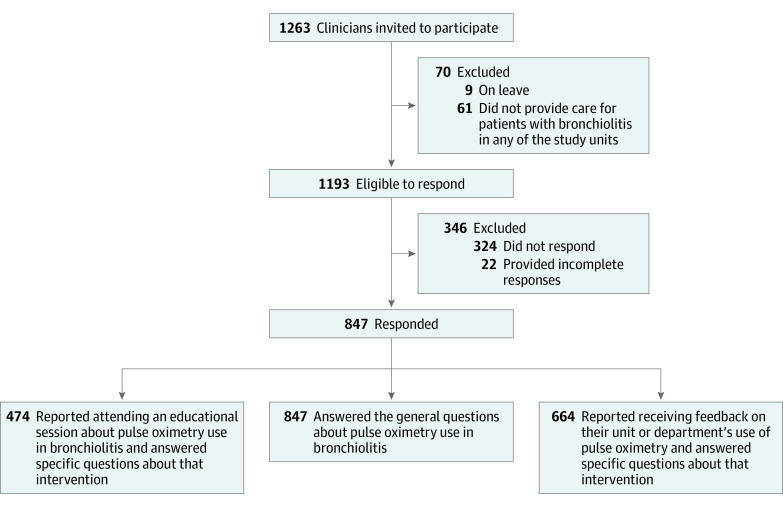
Questionnaire Participants Clinicians invited to participate included nurses, physicians, and respiratory therapists.

We distributed questionnaires to 1263 clinicians; 1193 clinicians were eligible and 847 responded, for an overall 71% response rate (range, 66%-84% among hospitals) (Figure 2). Of the 847 respondents, 474 reported attending at least 1 educational session, and 664 reported being provided with feedback data about their unit’s performance at least once; those respondents completed the corresponding questionnaire regarding acceptability, appropriateness, and feasibility. Additional details on response rates by profession are presented in eTable 1 in Supplement 2. Results are summarized in Table 1 and eTable 2 in Supplement 2.

**Table 1.** Results of Ordered Logistic Regression Model: Nurse and Physician Respondents

Questionnaire item	Nurses’ odds ratio of higher agreement than physicians (95% CI)	Difference between nurses and physicians in predicted marginal probability, % (95% CI)^a^
Audit and feedback acceptability		
I like the data feedback.	0.61 (0.36-1.03)	NA
I welcome continued data feedback about our use of cSpo_2_ in patients with bronchiolitis.	0.57 (0.33-1.00)	14 (<1-27)
Audit and feedback feasibility		
Data feedback about our use of cSpo_2_ in patients with bronchiolitis is easy to implement.	1.14 (0.75-1.74)	NA
Audit and feedback appropriateness		
Data feedback about our use of cSpo_2_ in patients with bronchiolitis seems like a good match for our non-ICU floors that care for patients with bronchiolitis.	0.72 (0.46-1.13)	NA
Education acceptability		
I like the educational sessions.	0.87 (0.57-1.34)	NA
I welcome continued educational sessions about appropriate use of cSpo_2_ in patients with bronchiolitis.	0.95 (0.58-1.54)	NA
Education feasibility		
Education sessions about the use of cSpo_2_ in patients with bronchiolitis are easy to implement on our non-ICU floors that care for patients with bronchiolitis.	1.06 (0.68-1.65)	NA
Education appropriateness		
Educational sessions about the appropriate use of cSpo_2_ in patients with bronchiolitis seem like a good match for our non-ICU floors that care for patients with bronchiolitis.	0.73 (0.52-1.02)	NA
Deimplementation intervention safety, norms, and intentions		
Intermittently spot-checking oxygen saturation instead of cSpo_2_ in stable, uncomplicated patients with bronchiolitis is safe.	0.28 (0.24-0.33)	30 (26-34)
Intermittently spot-checking oxygen saturation instead of cSpo_2_ in stable, uncomplicated patients with bronchiolitis is upsetting to parents.	2.38 (1.87-3.02)	11 (8-13)
Intermittently spot-checking oxygen saturation instead of cSpo_2_ in stable, uncomplicated patients with bronchiolitis is a good idea.	0.28 (0.24-0.32)	31 (27-34)
Intermittently spot-checking oxygen saturation instead of cSpo_2_ in stable, uncomplicated patients with bronchiolitis puts patients at risk.	3.91 (3.22-4.74)	21 (17-25)
Intermittently spot-checking oxygen saturation instead of cSpo_2_ in stable, uncomplicated patients with bronchiolitis could help us reduce length of stay.	0.30 (0.24-0.37)	29 (24-34)
Intermittently spot-checking oxygen saturation instead of cSpo_2_ in stable, uncomplicated patients with bronchiolitis could help us reduce monitor alarm fatigue.	0.70 (0.48-1.00)	NA
Most of my [nurse/physician] colleagues prefer intermittently spot-checking oxygen saturation instead of using cSpo_2_ in stable, uncomplicated patients with bronchiolitis.	0.40 (0.22-0.73)	20 (8-33)
Going forward, I intend to intermittently spot check oxygen saturation instead of using cSpo_2_ in stable, uncomplicated patients with bronchiolitis.	0.39 (0.34-0.46)	23 (19-27)

Abbreviations: cSpo_2_, continuous pulse oximetry; ICU, intensive care unit; NA, not applicable.

^a^ Included to assist in interpretation; reported only when *P* < .05.

### Educational Sessions

Respondents rated educational sessions favorably. Most agreed or completely agreed that they liked (435 of 474 [92%]) and welcomed (455 of 474 [96%]) the intervention (acceptability), that it was appropriate (457 of 474 [96%]), and that it was feasible (424 of 474 [89%]) (eTable 2 in Supplement 2). There were no significant differences between physician and nurse responses about the educational sessions (Table 1).

### Audit and Feedback

Respondents also rated the audit and feedback intervention favorably (eTable 2 in Supplement 2). Most agreed or completely agreed that they liked (615 of 664 [93%]) and welcomed (636 of 664 [96%]) the intervention (acceptability) and that the intervention was appropriate (622 of 664 [94%]) and feasible (557 of 664 [84%]). There were no significant differences between physician and nurse responses to questions about audit and feedback feasibility (Table 1). However, nurses had lower odds than physicians of agreeing that they welcomed continued feedback (a measure of acceptability; OR, 0.57; 95% CI, 0.33-1.00, *P* = .048).

### Acceptability, Feasibility, and Appropriateness

With respect to measures of appropriateness, perceived safety, norms, and intentions, most respondents agreed or completely agreed that intermittently spot-checking oxygen saturation instead of cSpo_2_ in stable patients with bronchiolitis was safe (803 of 847 [95%]), was a good idea (783 of 847 [92%]) and helps reduce length of stay (776 of 847 [92%]). Only 4% of respondents (37 of 847) agreed that intermittently spot-checking oxygen saturation instead of cSpo_2_ put patients at risk, and only 15% (129 of 847) agreed that intermittently spot-checking oxygen saturation instead of cSpo_2_ was upsetting to parents. Compared with physicians, nurses had lower odds of agreeing that intermittently spot-checking oxygen saturation instead of cSpo_2_ monitoring is safe (OR, 0.28; 95% CI 0.24-0.33; *P* < .001) and that it is a good idea (OR, 0.28; 95% CI, 0.24-0.32; *P* < .001). These interprofessional contrasts were driven by differences in responses of “completely agree” vs “agree” (eTable 2 in Supplement 2). Nurses had significantly higher odds of agreeing that intermittently spot-checking oxygen saturation instead of cSpo_2_ is upsetting to parents (OR, 2.38, 95% CI, 1.87-3.02; *P* < .001) and puts patients at risk (OR, 3.91; 95% CI, 3.22-4.74; *P* < .001). Nurses had lower odds of reporting that their nurse colleagues prefer intermittently spot-checking oxygen saturation instead of using cSpo_2_ monitoring (OR, 0.40; 95% CI, 0.22-0.73; *P* < .001) and lower odds of agreeing that they intend to intermittently spot-check oxygen saturation instead of using cSpo_2_ in stable patients with uncomplicated bronchiolitis going forward (OR, 0.39; 95% CI, 0.34-0.46; *P* < .001).

### Clinical Monitoring Use

Patient characteristics for the baseline and intervention periods are presented in Table 2. With respect to change in the practice of cSpo_2_, during the intervention period, 236 of 1051 patients (22%) with bronchiolitis who were not receiving supplemental oxygen received cSpo_2_ (hospital range, 4 of 47 [9%] to 110 of 255 [43%]) (Table 3), compared with 332 of 579 patients (57%) during the baseline period. Because of unit restructuring that occurred between the baseline and intervention periods (eg, changes in patient populations assigned to specific units and new units opening), directly comparing unit performance between the baseline and intervention periods was not possible. Using data from baseline period hospital units and adjusting for the same covariates used in the observational study’s analysis,^10^ guideline-discordant cSpo_2_ use decreased from 53% (95% CI, 49%-57%) to 23% (95% CI, 20%-25%; *P* < .001) during the intervention period (Table 3). The cSpo_2_ prevalence from the intervention period was 31 percentage points lower (95% CI, 26-35 percentage points) compared with baseline. This equates to a 31–percentage point increase in penetration^24,27^ of guideline-concordant care. There were no adverse events attributable to the intervention during the study.

**Table 2.** Patient Characteristics

Variable	Patient observations, No. (%)^a^	
	Baseline period (n = 579)	Intervention period (n = 1051)
**Patient demographics**		
Age		
8 wk-5 mo	281 (49)	534 (51)
6-11 mo	160 (28)	284 (27)
12-17 mo	89 (15)	153 (15)
18-23 mo	49 (9)	80 (8)
Gestational age		
Preterm (28 0/7 to 33 6/7 wk)	55 (10)	85 (8)
Not preterm^b^	524 (91)	966 (92)
Sex		
Male	349 (60)	611 (58)
Female	230 (40)	438 (42)
Not specified	0	2 (0.2)
Race^c^		
White	226 (39)	354 (34)
Black or African American	123 (21)	236 (23)
Specified as other	155 (27)	370 (35)
Specified as unknown	30 (5)	32 (3)
Asian	35 (6)	38 (4)
>1 race	8 (1)	15 (1)
Native Hawaiian or Pacific Islander	0	5 (0.5)
American Indian or Alaska Native	2 (0.3)	1 (0.1)
Ethnicity^c^		
Not Hispanic or Latino	337 (58)	609 (58)
Hispanic or Latino	172 (30)	305 (29)
Unknown	62 (11)	130 (12)
Other	8 (1)	7 (1)
**Illness characteristics**		
Time since weaning from supplemental oxygen, h		
Never received	226 (39)	393 (37)
<1	7 (1)	29 (3)
1 to <2	13 (2)	29 (3)
2 to <4	25 (4)	55 (5)
4 to <6	28 (5)	64 (6)
6 to <12	78 (14)	140 (13)
12 to <24	116 (20)	185 (18)
≥24	84 (15)	156 (15)
Unknown	2 (0.3)	0
Apnea or cyanosis^d^	33 (6)	39 (4)
Comorbid condition associated with neurological impairment^e^	21 (4)	23 (2)
Enteral feeding tube (nasogastric or gastrostomy)	67 (12)	80 (8)
Hospital type		
Freestanding children’s hospital (n = 5)	522 (90)	1004 (96)
Community hospital (n = 1)	57 (10)	47 (5)
Observation performed during “overnight” hours (11 pm to 7 am)	272 (47)	107 (10)

^a^ For some variables, the sum of percentages does not equal 100% because of rounding.

^b^ Not preterm included the following: documented gestational age 34 0/7 weeks and above, absence of gestational age but documented as full term, or absence of gestational age but not labeled in medical record as preterm or premature.

^c^ Patient family-reported race and ethnicity were abstracted from charts in categories defined by the Standards for the Classification of Federal Data on Race and Ethnicity, in compliance with National Institutes of Health inclusion reporting policies.

^d^ Includes documentation of apnea or cyanosis occurring at home or in the hospital during the present illness.

^e^ Static encephalopathy, cerebral palsy, hydrocephalus, spina bifida, epilepsy or seizure disorder, or hypotonia.

**Table 3.** Continuous Spo_2_ Use Rates in Patients Not Receiving Supplemental Oxygen (Guideline-Discordant Monitoring)

Hospital	Baseline period		Intervention period		Adjusted percentage decrease in monitored patients after the intervention, (95% CI)
	Monitored observations, No. (%)	Adjusted percentage monitored, (95% CI)	Monitored observations, No. (%)	Adjusted percentage monitored, (95% CI)	
A	49/70 (70)	65 (54-76)	24/88 (27)	30 (21-39)	35 (20-49)
B	40/57 (70)	69 (56-83)	4/47 (9)	9 (1-16)	61 (46-76)
C	62/95 (65)	63 (54-73)	30/161 (19)	20 (14-25)	44 (32-55)
D	72/111 (65)	66 (58-74)	28/97 (29)	27 (19-35)	39 (27-50)
E	36/154 (23)	22 (16-28)	40/403 (10)	10 (7-13)	12 (5-18)
F	73/92 (79)	78 (70-87)	110/255 (43)	43 (36-50)	35 (25-46)
Overall	332/579 (57)	53 (49-57)	236/1051 (22)	23 (20-25)	31 (26-35)

Abbreviation: Spo_2_, pulse oximetry.

## Discussion

In this 6-hospital single-group cSpo_2_ deimplementation trial using historical control data, most respondents agreed that the deimplementation strategies targeting guideline-discordant cSpo_2_ for patients with bronchiolitis not receiving supplemental oxygen were acceptable, appropriate, feasible, and safe. Application of these strategies was temporally associated with a significant decrease in the adjusted percentage of hospitalized children with bronchiolitis not receiving supplemental oxygen who received guideline-discordant cSpo_2_.

We noted important interprofessional differences in perceptions of intervention safety between nurses and physicians, which warrant further study and have implications for future deimplementation efforts. The deimplementation of practices considered safe in children may be particularly challenging.^32^ Continuous physiological monitoring has been widely adopted into clinical surveillance in various settings based on a common belief that it improves safety.^33^ Nurses in adult settings report that continuous physiological monitoring of patients outside of the intensive care unit improves patient safety.^34^ We found significant differences in the perceived safety of intermittently spot-checking oxygen saturation, with nurses rating this approach significantly lower than physician participants. The contrast was driven by differences in the distributions of “completely agree” vs “agree” responses, however, which may or may not be clinically important. This observed difference may relate to the scope of practice for nurses in pediatric hospital settings, where advocating for clinical decisions regarding the escalation or de-escalation of physiological monitoring to improve the detection of clinical deterioration is common.^35^ Furthermore, the education and audit and feedback interventions were delivered to each professional in the same format in this study. It is possible that tailoring the audit and feedback strategy to include clinicians setting role-specific goals, providing clinician-concordant benchmarks, and ensuring role concordance of the person delivering the feedback may improve perceptions of safety. Although clinicians overall rated the intervention favorably, the differences we observed in nurses’ perception of safety will need to be a focus of future deimplementation work in this area.

Although our findings suggest that audit and feedback and education are associated with positive clinician perceptions and deimplementation of cSpo_2_, prior studies suggest that decay in improvement can occur after they are removed.^36^ A follow-up study of a multicenter learning collaborative found that many interventions were not sustained after the intervention period ended.^37,38^ Educational outreach strategies declined in use from 73% to 37% of hospitals, and data audits declined from 88% to 30%, with respondents citing insufficient time and competing priorities. These findings are consistent with the perspective that education-based interventions are a necessary component in multistrategy interventions but rarely result in sustained behavior changes alone.^39^ Education may require fewer resources to implement than audit and feedback, as suggested by the high feasibility ratings in our study. Future studies can elucidate mechanisms that contribute to the effectiveness of differing configurations, including the ideal “dose” of audit and feedback and educational outreach, and anticipate necessary adaptations depending on the setting.

The specific aspects of the education and audit and feedback strategies’ association with cSpo_2_ deimplementation in children’s hospitals are not well-established. Three single-center quality improvement studies used audit and feedback or educational outreach alongside additional components, such as the use of champions, standard pathways, and order set modifications.^14,16,17^ A multicenter improvement study using education along with capacity building saw increases in intermittent pulse oximetry orders.^15^ Although these studies describe improvements in practice, most used multiple concurrent or sequential interventions with less focus on evaluating distinct strategies. Based on the generally favorable clinician perceptions, our findings support further testing of audit and feedback and educational outreach to address cSpo_2_ use for hospitalized patients with bronchiolitis. Additional interventions, such as champions and electronic health record–based interventions, should be assessed in future studies to determine whether they are associated with deimplementation.

### Limitations

This study has several limitations. First, 5 of the 6 study hospitals were freestanding children’s hospitals, and all participated in the baseline measurement study^10^ in which some clinicians may have been exposed to education. Our findings may not be generalizable to all inpatient settings where patients with bronchiolitis receive care. Second, we used a convenience sampling approach for the monitoring practice audits, which may have resulted in a nonrepresentative sample. Third, our questionnaire response rate of 71% was adequate, but it remains possible that those with more negative experiences with the intervention chose not to participate, biasing our findings toward more positive views of deimplementation. Fourth, the historical controls were collected in the bronchiolitis season immediately preceding this study’s intervention period. It is possible that secular trends occurring in the months between bronchiolitis seasons may have influenced monitoring practices and clinician perceptions of the deimplementation intervention.

## Conclusions

Educational outreach and audit and feedback deimplementation strategies were temporally associated with reductions in cSpo_2_ use during the intervention period. Clinicians rated these interventions favorably in terms of acceptability, appropriateness, feasibility, and perceived safety, suggesting that these are sensible strategies to begin addressing guideline-discordant cSpo_2_ use. Further studies should focus on how best to sustain improvements brought about by personnel-intensive interventions, such as educational outreach and audit and feedback, and seek to mechanistically understand key interprofessional, contextual, and strategy-specific characteristics associated with deimplementation success.

## Notes

Supplement 1.
Trial Protocol

Supplement 2. **eTable 1.** Questionnaire Response Rates by Site and Role **eTable 2.** Clinician Questionnaire Responses **eFigure 1.** Sample Clinician Educational Material **eFigure 2.** Sample Feedback Dashboard **eAppendix.** Questionnaire

Supplement 3.
Nonauthor Collaborators

Supplement 4.
Data Sharing Statement
