# Supplementary material for: Evaluation of an Educational Outreach and Audit and Feedback Program to Reduce Continuous Pulse Oximetry Use in Hospitalized Infants With Stable Bronchiolitis: A Nonrandomized Clinical Trial
Source: JAMA Netw Open. 2021 Sep 2;4(9):e2122826. doi: 10.1001/jamanetworkopen.2021.22826 (PMC8414187; doi:10.1001/jamanetworkopen.2021.22826)
Supplement: Supplement 1. — Trial Protocol [file jamanetwopen-e2122826-s001.pdf]

1

Title:	**Feasibility pilot of audit and feedback with educational outreach to align continuous pulse oximetry use in stable bronchiolitis patients with evidence and guideline recommendations**
Short Title	Aligning pulse oximetry with guidelines
eIRB Number	19-016718
Protocol Date:	8/17/2019
Amendment 1 Date:	Amendment 3 Date:
Amendment 2 Date:	Amendment 4 Date:

<u>Study Principal Investigator</u>

Christopher P. Bonafide, MD, MSCE
Children's Hospital of Philadelphia
3500 Civic Center Blvd

Buerger Building 12th Floor
Philadelphia, PA, 19104
Phone 267-426-2901
email: bonafide@chop.edu

2

TABLE OF CONTENTS

Table of Contents .... **ii**
Abbreviations and Definitions of Terms .... **iv**
Abstract .... **v**

1 BACKGROUND INFORMATION AND RATIONALE **1**

1.1 Introduction 1
1.2 Relevant Literature and Data 1
1.2.1 Bronchiolitis SpO_2 monitoring guidelines *1*
1.2.2 Research published since bronchiolitis SpO_2 monitoring guidelines *1*
1.2.3 Unpublished trials of continuous versus intermittent SpO_2 monitoring in bronchiolitis *2*
1.2.4 Results of Best Evidence for Effective Pediatric (BEEP) Monitoring Study *2*
1.2.5 Measurement of pulse oximetry use using direct observation during the 2018-19 bronchiolitis season *3*
1.2.6 Effective strategies for implementing practice guidelines *4*
1.3 Compliance Statement 4

2 STUDY OBJECTIVES **4**

2.1 Primary Objective (or Aim) 4
2.2 Secondary Objectives (or Aim) 4

3 INVESTIGATIONAL PLAN **4**

3.1 General Schema of Study Design 4
3.2 Allocation to Treatment Groups and Blinding 5
3.3 Study Duration, Enrollment and Number of Sites 5
3.3.1 Duration of Study Participation *5*
3.3.2 Total Number of Study Sites/Total Number of Subjects Projected *5*
3.4 Study Population 6
3.4.1 Inclusion Criteria *6*
3.4.2 Exclusion Criteria *6*

4 STUDY PROCEDURES **7**

4.1 Pre-intervention period 7
4.2 Intervention period 7
4.3 At conclusion of the intervention period 7

5 STUDY EVALUATIONS AND MEASUREMENTS **7**

5.1 Overview 7
5.2 Chart review 8
5.2.1 Site investigators will provide the following contextual information about audit and feedback and educational outreach delivery events, using a framework previously described:[29] *8*
5.2.2 The following data elements will be abstracted from the chart and stored in the study database:8
5.2.3 The following data elements will be abstracted from the chart and stored in the individual site's separate master list alongside the coded ID, admission date, and data collection rounds date but NOT entered into the study database and NOT transmitted outside of the institution where the patient is hospitalized: *9*
5.3 Bedside visual observation "rounds" 10
5.4 Staff questionnaires (see appendix for instrument) 10
5.4.1 Site PIs will submit to CHOP for recruitment *10*
5.4.2 The questionnaires will include the following elements: *10*

6 STATISTICAL CONSIDERATIONS **10**

6.1 Primary Endpoint 10

6.2 SECONDARY ENDPOINTS 10
6.3 STATISTICAL METHODS 11
6.3.1 Questionnaire-based methods (AIM, IAM, FIM) 11
6.3.2 Pulse oximetry use rates 11
6.3.3 Medical device integration test characteristics 11
6.4 SAMPLE SIZE AND POWER 11

7 SAFETY MANAGEMENT 11

7.1 CLINICAL ADVERSE EVENTS 11
7.2 ADVERSE EVENT REPORTING 11

8 STUDY ADMINISTRATION 11

8.1 TREATMENT ASSIGNMENT METHODS 11
8.2 DATA COLLECTION AND MANAGEMENT 12
8.2.1 Data sources 12
8.3 CONFIDENTIALITY 12
8.4 REGULATORY AND ETHICAL CONSIDERATIONS 12
8.4.1 Data and Safety Monitoring Plan 12
8.4.2 Risk Assessment 14
8.4.3 Potential Benefits of Trial Participation 14
8.4.4 Risk-Benefit Assessment 14
8.5 RECRUITMENT STRATEGY 14
8.6 INFORMED CONSENT/ASSENT AND HIPAA AUTHORIZATION 15
8.6.1 Informed consent and HIPAA for the primary measure (questionnaires) 15
8.6.2 Waiver of Consent and Assent for all other aspects of the study 15
8.6.3 Waiver of Assent 16
8.6.4 Waiver of HIPAA Authorization 16
8.7 PAYMENT TO SUBJECTS/FAMILIES 16

9 PUBLICATION 17

10 APPENDIX 18

10.1 CONSENT LANGUAGE FOR QUESTIONNAIRE 18
10.1.1 Text listed on questionnaire itself .... **Error! Bookmark not defined.**
10.1.2 Text listed on attached/linked document .... **Error! Bookmark not defined.**
Yes. Completion of the questionnaire below indicates your willingness to participate and serves as your consent. .... **Error! Bookmark not defined.**
10.2 DRAFT QUESTIONNAIRE ITEMS 18

11 REFERENCES 19

88 ABBREVIATIONS AND DEFINITIONS OF TERMS

AAP	American Academy of Pediatrics
AIM	Acceptability of Intervention Measure
BEEP	Best Evidence for Effective Pediatric Monitoring Study
BIDS	Bronchiolitis of Infancy Discharge Study
EHR	Electronic health record
FIM	Feasibility of Intervention Measure
IAM	Intervention Appropriateness Measure
ICU	Intensive care unit
PRIS	Pediatric Research in Inpatient Settings Network
SpO_2	Pulse oximetry

ABSTRACT

Context:

Continuous pulse oximetry (SpO_2) monitoring has revolutionized detection of oxygen desaturation in operating rooms and other high-risk areas, improving outcomes in those settings.[1,2] However, research suggests that overuse of continuous SpO_2 monitoring in stable children with bronchiolitis who are unlikely to benefit from it is low-value care that places some children at risk of adverse outcomes.[3–12] Despite national guidelines[13,14] discouraging continuous pulse oximetry use in stable bronchiolitis patients, 46% of those infants and children are continuously monitored.

Objectives:

The primary objective is to measure the feasibility, acceptability, and appropriateness of audit and feedback with educational outreach as a strategy to align continuous pulse oximetry use in stable bronchiolitis patients with evidence and guideline recommendations.

Study Design:

Pragmatic, prospective, non-randomized, single-arm feasibility pilot implementation with the intervention delivered at the hospital unit level.

Setting/Participants:

The pilot will be performed on non-ICU hospital units that care for bronchiolitis patients at up to 10 total hospitals in the United States. The primary subjects are hospital staff who order or manage continuous pulse oximetry monitoring for bronchiolitis patients. Secondary subjects are patients age 2 through 23 months with a primary diagnosis of bronchiolitis.

Study Interventions and Measures:

The intervention is an audit and feedback strategy that includes providing the hospital’s own continuous pulse oximetry use data back to them on a weekly basis. The data will be accompanied by staff-targeted educational materials and outreach sessions summarizing the current evidence and guideline recommendations for continuous pulse oximetry use in bronchiolitis.

Primary measurements include measures of feasibility, acceptability, and appropriateness of the audit and feedback strategy. Secondarily, we will analyze continuous pulse oximetry use rates and validate an alternative semi-automated data collection strategy.

1 BACKGROUND INFORMATION AND RATIONALE

1.1 Introduction

Continuous pulse oximetry (SpO_2) monitoring has revolutionized detection of oxygen desaturation in operating rooms and other high-risk areas, improving outcomes in those settings.[1,2] However, research suggests that overuse of continuous SpO_2 monitoring in stable children with bronchiolitis who are unlikely to benefit from it is low-value care that places some children at risk of adverse outcomes.[3–12]

Despite national guidelines[13,14] discouraging continuous pulse oximetry use in stable bronchiolitis patients, 46% of those infants and children are continuously monitored (not yet published data from a multicenter study performed in 56 hospitals led by investigators on this proposal).

This study aims to pilot an audit and feedback strategy to align continuous pulse oximetry use in stable bronchiolitis patients with evidence and guideline recommendations in preparation for a multicenter cluster-randomized hybrid trial.

1.2 Relevant Literature and Data

1.2.1 Bronchiolitis SpO_2 monitoring guidelines

Appropriate use of SpO_2 monitoring in children with bronchiolitis is guided by the American Academy of Pediatrics (AAP) Clinical Practice Guideline for the Diagnosis, Management, and Prevention of Bronchiolitis[13] and Society of Hospital Medicine Choosing Wisely recommendations.[14] The AAP Guideline was published by Ralston (Advisory Committee member) and colleagues in 2014.[13] The Guideline states that *"Clinicians may choose not to use continuous pulse oximetry for children with a diagnosis of bronchiolitis,"* citing intermittent monitoring benefits including shorter length of stay, decreased alarm fatigue, and decreased cost. The Society of Hospital Medicine Choosing Wisely in Pediatric Hospital Medicine workgroup took a stronger stance against monitoring, stating *"Do not use continuous pulse oximetry routinely in children with acute respiratory illness unless they are on supplemental oxygen."*[14]

1.2.2 Research published since bronchiolitis SpO_2 monitoring guidelines

Since publication of the AAP Guideline and Choosing Wisely recommendations, 3 randomized trials,[3–5] 2 nonrandomized studies,[6,7] and 1 expert commentary[15] have *further strengthened the evidence base against using continuous SpO_2 monitoring in favor of intermittent SpO_2 measurement in children with bronchiolitis not requiring supplemental oxygen.* The research below demonstrates that intermittent SpO_2 monitoring is a safe alternative,[3,7] those who are continuously monitored unnecessarily are at risk of adverse outcomes including prolonged length of stay,[5,8,9] iatrogenic harm,[10] and increased costs,[5] and false SpO_2 monitor alarms contribute to alarm fatigue.[11,12] McCulloh et al performed a randomized trial comparing continuous versus intermittent SpO_2 monitoring among children with bronchiolitis at 4 hospitals who had stable oxygen saturations of 90% or higher off supplemental oxygen.[3] They found that patients who underwent intermittent SpO_2 measurement did not have increased urgent care escalation or respiratory interventions, confirming its safety.

Schuh et al performed a randomized trial among infants presenting to an emergency department with bronchiolitis.[4] Infants were randomized to have their SpO_2 measured either with an oximeter with true SpO_2 displayed or a modified oximeter with falsely elevated SpO_2 displayed (+3 percentage points). Patients whose SpO_2 was artificially elevated were less likely to be hospitalized but had no significant differences in other clinical outcomes, including no difference in subsequent unscheduled follow up visits for bronchiolitis. We interpret these findings as indicating that continuous pulse oximetry likely identifies minor desaturations that do not impact outcomes but are over weighted in clinical decision making (in this study, the decision to admit).

In the Bronchiolitis of Infancy Discharge Study (BIDS), a randomized controlled trial of hospitalized infants with bronchiolitis, Cunningham et al randomized infants to be continuously monitored using either a standard pulse oximeter or a modified oximeter that falsely inflated SpO_2, displaying a measured value of 90% as 94%.[5] They found that supplemental oxygen was provided less often and

for shorter periods of time in the group with SpO_2 values falsely inflated, an expected outcome. However, they also found that infants were discharged 10 hours sooner in the group with SpO_2 values falsely inflated, and yet there were no differences in any adverse effects of the falsely inflated oxygen saturation value, including readmission or time until return to daycare. In an economic analysis of BIDS, the standard oximeter group had significantly greater hospital costs than the modified group, primarily driven by costs of the hospital bed, supplemental oxygen, and oximeter probes.[16] Like Schuh's study, we interpret these findings as indicating that continuous pulse oximetry likely identifies minor desaturations that do not impact patient outcomes but are over weighted in clinical decision making, unnecessarily prolonging oxygen administration and length of hospital stay and increasing costs.

Principi et al performed a cohort study of infants presenting to an emergency department with bronchiolitis who were well enough to be discharged home.[6] Prior to discharge, researchers applied pulse oximeters to the infants. The oximeters had alarms disabled and their displays did not show SpO_2 values. They collected the oximeters 72 hours later. They found that half of these infants deemed well enough for discharge had desaturations to 80% or less for at least 1 minute and one-quarter had desaturation to 70% or less for at least 1 minute. There were no differences in rates of return medical visits or subsequent hospitalizations between infants who experienced desaturations and those who did not. We interpret these findings as indicating that brief desaturations to levels that would likely have been treated if they occurred in hospital are common in patients with bronchiolitis at home, but are not associated with adverse outcomes.

Schondelmeyer et al evaluated a quality improvement intervention targeting reduction of continuous pulse oximetry in children with bronchiolitis and asthma when they no longer required supplemental oxygen.[7] Their intervention included staff education, checklists, and electronic health record clinical decision support. They found a reduction of median time on continuous pulse oximetry from 10.7 to 3.1 hours without a difference in rapid response team activations, ICU transfers, or readmissions. This study confirms the safety of the AAP Guideline and Choosing Wisely recommendations, and supports the practice of removing continuous pulse oximetry after supplemental oxygen is discontinued and replacing it with intermittent SpO_2 monitoring.

In a 2017 BMJ expert commentary, "When technology creates uncertainty: pulse oximetry and overdiagnosis of hypoxaemia in bronchiolitis,"[15] Quinonez et al reviewed the literature above and argue that the widespread use of pulse oximetry in stable bronchiolitis patients leads to overdiagnosis of hypoxemia. Overdiagnosis is when a true abnormality is discovered, but awareness of the abnormality does not benefit the patient.[17] The authors conclude that "current body of evidence suggests that we should challenge assumptions regarding the detection and aggressive management of borderline hypoxemia in non-critically ill infants with bronchiolitis," because current overuse of continuous pulse oximetry has led to overdiagnosis of clinically insignificant hypoxemia that contributes to unnecessary resource utilization and risk of patient harm.

1.2.3 Unpublished trials of continuous versus intermittent SpO_2 monitoring in bronchiolitis

We identified 2 randomized controlled trials of continuous versus intermittent SpO_2 monitoring in bronchiolitis led by Mahant in Toronto. The first trial is a single center pilot that has been completed.[18] The second is a 6-center trial that is ongoing.[19] While results of the intervention on the primary outcome (length of stay) are not yet available, in a personal communication the Principal Investigator reported that among the 89 patients randomized to intermittent (as opposed to continuous) SpO_2 monitoring to date, none have experienced adverse safety events attributable to the less intensive intermittent monitoring intervention.[20]

1.2.4 Results of Best Evidence for Effective Pediatric (BEEP) Monitoring Study

The BEEP study, performed in 2018 (not yet published) and led by Amanda Schondelmeyer, MD, MSc used the RAND/UCLA Appropriateness method and a panel of experts nominated by leaders of national nursing, physician, human factors, and respiratory therapy organizations as well as a family advocacy organization to develop consensus guidelines for physiologic monitoring in hospitalized children in non-ICU settings. Pertinent to this proposal, the expert panel determined the following scenarios to be appropriate and necessary:

- *Children hospitalized with routine bronchiolitis and an SpO_2 value of >=90% without supplemental oxygen in the absence of witnessed apnea or cyanosis should NOT receive continuous pulse oximetry monitoring, instead they should receive intermittent pulse oximetry measurement*
- *Children weaned from supplemental oxygen should be transitioned to intermittent oximetry measurement within 1 hour if oxygen saturations are stable at >=90% unless otherwise indicated by diagnosis or condition.*

1.2.5 Measurement of pulse oximetry use using direct observation during the 2018-19 bronchiolitis season

From December 2018 through March 2019, our team performed a 56-center observational study within the Pediatric Research in Inpatient Settings (PRIS) Network measuring pulse oximetry use in stable infants and children not requiring supplemental oxygen.[21] This is the population for whom the AAP and Choosing Wisely support avoiding the use of continuous pulse oximetry.

The study aimed to include exclusively patients for whom continuous pulse oximetry is not indicated according the AAP and Choosing Wisely guidelines: Children 8 weeks-23 months old with primary diagnosis of bronchiolitis on most recent physician note, hospitalized on generalist services in non-ICU areas, and not currently on any supplemental oxygen or room air flow support. We excluded infants born at <28 weeks, those receiving home oxygen or positive pressure ventilation, and those with major cardiac, respiratory, neuromuscular, immunologic, and oncologic comorbidities. This project was funded by NHLBI and was approved by the CHOP IRB as the reviewing IRB (CHOP IRB# 18-015070).

In this study that included over 3600 in-person assessments of pulse oximetry monitoring status, we found that the overall monitoring rate across all hospitals was 46%, with a very wide range of variation, ranging from hospitals that monitor <5% of these patients to hospitals that monitor >85% of these patients (results not yet published; see figure below). These results support efforts to reduce continuous pulse oximetry in stable bronchiolitis patients in outlier hospitals with high use rates.

Prior to submitting a subsequent grant that will aim to de-implement continuous pulse oximetry in a well-defined stable bronchiolitis population, we must establish the feasibility of a foundational audit and feedback strategy for addressing continuous pulse oximetry overuse.

Continuous pulse oximetry overuse rates
(by hospital, unadjusted, with 95% CIs)

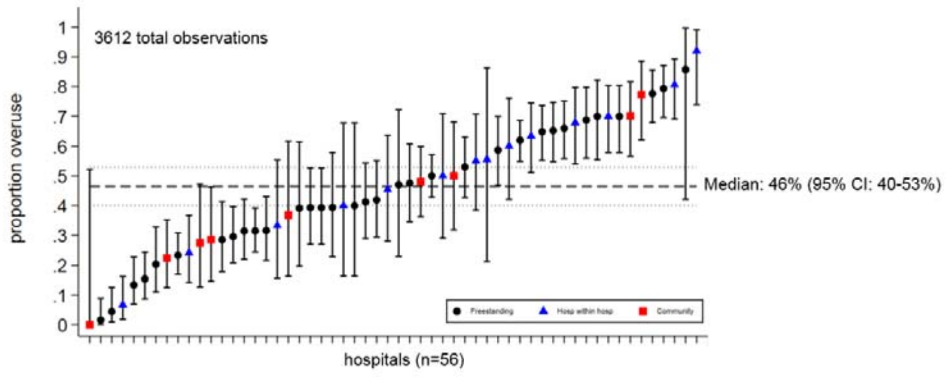

1.2.6 Effective strategies for implementing practice guidelines

In a recent report from the American College of Cardiology/American Heart Association Task Force on Clinical Practice Guidelines, evidence from the published implementation science literature was presented describing effective strategies to enhance the adoption and implementation of clinical practice guidelines.[22] The review concluded that audit and feedback and educational outreach strategies were the most effective in improving outcomes measuring processes of care as well as clinical outcomes. However, the specific aspects of audit and feedback that make this strategy effective (e.g. who the feedback data are delivered to, what information is delivered, is comparison data provided, are data presented graphically, what is the lag time between data collection and feedback, etc) are not well established, but are an active area of inquiry in the field of implementation science. The subsequent trial that we will plan based upon the results of this pilot will secondarily aim to elucidate mechanisms that contribute to the effectiveness of differing configurations of audit and feedback.

1.3 Compliance Statement

This study will be conducted in full accordance with all applicable Children's Hospital of Philadelphia Research Policies and Procedures and all applicable Federal and state laws and regulations. All episodes of noncompliance will be documented.

The investigators will perform the study in accordance with this protocol and will report unanticipated problems involving risks to subjects or others in accordance with Children's Hospital of Philadelphia IRB Policies and Procedures and all federal requirements. Collection, recording, and reporting of data will be accurate and will ensure the privacy, health, and welfare of research subjects during and after the study.

2 STUDY OBJECTIVES

2.1 Primary Objective (or Aim)

The primary objective of this study is to measure the feasibility, acceptability, and appropriateness of audit and feedback with educational outreach as a strategy to align continuous pulse oximetry use in stable bronchiolitis patients with evidence and guideline recommendations.

2.2 Secondary Objectives (or Aim)

The secondary objectives are to:

- Measure continuous pulse oximetry use rates in children with bronchiolitis who are not requiring supplemental oxygen administration.
- *In hospitals with "medical device integration"* in which data from physiologic monitors are sent directly to the electronic health records, we will:
    - Validate that the medical device integration data in the electronic health record reflects the findings of direct in-person observation described above as the gold standard.

3 INVESTIGATIONAL PLAN

3.1 General Schema of Study Design

This is a pragmatic,[23] prospective, non-randomized, single-arm feasibility pilot implementation with the audit and feedback plus educational outreach intervention delivered at the hospital unit (i.e. ward) level.

This study does not directly involve making any changes to patient care – it only involves presenting data, guidelines, and evidence to clinical staff who make independent decisions about their own actions related to pulse oximetry monitoring in bronchiolitis. Any individual clinicians who do not wish

to participate may choose not to interact with the electronic communications and may choose to not attend meetings where this intervention is discussed.

On the Pragmatic-Explanatory Continuum,[23] this study is highly pragmatic for the following reasons (see cited article for detailed explanation of domains):

- Eligibility criteria: The same bronchiolitis patients who would likely be a candidate for the audit and feedback intervention if it was being provided in usual care are also eligible for this intervention since it is applied at the hospital unit level.
- Recruitment: The audit and feedback intervention will be delivered to the same health care professionals who are involved in the decisions or actions surrounding the initiation or discontinuation of continuous pulse oximetry monitoring in usual care.
- Setting: The pilot is being performed in hospital settings that are identical to the types of settings where we intend the results to be applied.
- Organization: Given the highly pragmatic nature of this study, the intervention will be delivered into each hospital using the same structures of care used locally for making improvements in bronchiolitis care delivery.
- Flexibility in intervention delivery: Consistent with the idea that interventions can be tailored to local context, we will allow for flexibility/adaptation/tailoring in the content and format of the educational materials and outreach sessions. Thus, the methodology of exactly how to deliver this intervention is not rigidly prescriptive in this protocol. In addition, it is possible that some sites will be performing concurrent quality improvement interventions targeting pulse oximetry overuse. We will seek to fully understand when and how any potential quality improvement interventions interact with this study but we will not restrict sites from performing separate improvement initiatives nor will we will regulate or oversee their concurrent quality improvement projects.
- Flexibility in adherence to the intervention: While we will monitor health care professionals' engagement with the interventions and the resulting pulse oximetry use that results at the patient level, as in usual care, there are no special measures in place beyond what would occur in the course of a practice change in the usual care of bronchiolitis.

3.2 Allocation to Treatment Groups and Blinding

N/A: This is a single-arm study.

3.3 Study Duration, Enrollment and Number of Sites

The intervention period will last between 2-4 months depending on the timing relative to the local epidemiology-dictated bronchiolitis season.

The primary subjects are hospital staff. Secondary subjects are patients age 2 through 23 months with a primary diagnosis of bronchiolitis.

3.3.1 Duration of Study Participation

- Hospital staff: 2-4 months.
- Patients: minimum of 1 day, maximum of the duration of their entire hospitalization (maximum 4 months; anticipated average of 1-3 days).

3.3.2 Total Number of Study Sites/Total Number of Subjects Projected

The study will be conducted in up to 10 hospitals in the United States.

Primary subjects exposed to the intervention (hospital staff): Since the intervention is occurring at a unit-wide level and we are not collecting any identifiers on these primary subjects, it is not possible to accurately measure the actual number of staff whose behavior could potentially have been impacted by the intervention. It also will not be possible to identify when hospital staff are exposed to the intervention repeatedly over time. In order to best estimate this number, we will "count" the

involvement of 100 hospital staff per unit during the entire intervention period. Based on an average of 5 available units per hospital in candidate hospitals, we can estimate 10 hospitals * 5 units per hospital * 100 staff per unit = 5,000 hospital staff subjects, acknowledging that this number will not be completely accurate but is a reasonable estimate. When we report the “actual” number of primary exposed subjects involved for continuing reviews and study closure, we will report 100 * the total number of units involved in the study.

<u>Primary subjects completing the questionnaires (hospital staff):</u> We will aim for 100 completed questionnaires per hospital x 10 hospitals = 1,000 primary subjects completing the questionnaires. Since these 1,000 will be drawn from within the 5,000 listed above, they will not add to the overall subject total.

<u>Secondary subjects - Patients:</u> We project collecting data on a maximum of 300 unique bronchiolitis patients per hospital * 10 hospitals = 3,000 patients.

3.4 Study Population

3.4.1 Inclusion Criteria

3.4.1.1 <u>Primary subjects - Hospital staff</u>

1) Nurses, physicians, physician assistants, nursing assistants, or respiratory therapists
2) Providing care to patients on units included in the study.

3.4.1.2 <u>Secondary subjects - Patients</u>

1) Males or females 8 weeks through 23 months old hospitalized on non-ICU wards
2) Cared for by generalist inpatient services (e.g. general pediatrics, hospital medicine)
3) Primary diagnosis of acute bronchiolitis according to hospital chart
4) Not currently requiring supplemental oxygen therapy or nasal cannula flow at an FiO_2 of 21% (room air flow)

3.4.2 Exclusion Criteria

3.4.2.1 <u>Primary subjects - Hospital staff</u>

1) None. Staff may self-exclude by choosing not to interact with the electronic communications and may choose to not attend meetings where the intervention is discussed.

3.4.2.2 <u>Secondary subjects - Patients</u>

1) Premature birth: <28 weeks completed gestation
2) Cyanotic congenital heart disease
3) Pulmonary hypertension
4) Home oxygen or positive pressure ventilation requirement
5) Tracheostomy
6) Neuromuscular disease
7) Immunodeficiency
8) Cancer

Subjects that do not meet all of the enrollment criteria may not be enrolled. Any violations of these criteria must be reported in accordance with IRB Policies and Procedures.

4 STUDY PROCEDURES

4.1 Pre-intervention period

- Implementation champions identified, local teams formed
- Local team members will meet with leadership on each potential unit; unit leadership will be given the opportunity to opt their unit out of the study for any reason.
- Implementation champions & teams perform educational outreach sessions on participating units
    - describing current guidelines
    - summarizing the evidence behind the guidelines
    - discussing potential alternatives to continuous pulse oximetry monitoring for patients in whom guidelines do not advise continuous pulse oximetry
- Distribution/dissemination of educational materials (e.g. slide presentations, posters, flyers, videos) to hospital staff working on study units focused on the bullet points above
    - materials collaboratively developed by the CHOP team and local implementation teams, with site-specific tailoring by the local implementation teams

4.2 Intervention period

- Implementation teams will conduct data collection rounds and supplemental chart review (see Section 5) to determine continuous pulse oximetry status on bronchiolitis patients not receiving supplemental oxygen or room air flow, targeting data collection at least twice per week in order to acquire sufficient data for audit and feedback intervention.
- If any hospital staff responsible for the care of the patient are present and available on the unit, the data collector may perform real-time inquiry if feasible, briefly asking about the indications for monitoring individual patients, which will be documented in the study database as field notes without documenting any of the hospital staff member's identifiers.
- On a weekly basis, the data will be summarized in a dashboard and the dashboard along with the raw data will be returned to the site PI.
- Local implementation team will then share that data in electronic and in-person settings appropriate and tailored to each site (e.g. email, safety huddles,[24,25] staff meetings, rounds, staff messages in electronic health record systems) with key clinician stakeholders who order or manage continuous pulse oximetry monitoring for bronchiolitis patients. Data will be contextualized with (a) the hospital's prior performance, (b) other hospitals' prior performance, (c) the current performance of other units in the same hospital, (d) the current performance of other units in other hospitals.
- Additional educational materials will be distributed and additional education/outreach sessions will be held by the local implementation team at a frequency determined by the site PI depending on local pulse oximetry use rates.

4.3 At conclusion of the intervention period

- Administer questionnaire-based psychometrically valid measures to hospital staff assessing feasibility, acceptability, appropriateness,[26] and perceived safety of the audit and feedback strategy.

5 STUDY EVALUATIONS AND MEASUREMENTS

5.1 Overview

Data collection will be operationalized at sites during periodic in-person observational "rounds" during the intervention period. During a data collection round, site investigators will first identify all

patients currently admitted to their hospital's non-ICU pediatric wards with bronchiolitis by reviewing the census of each unit that cares for children with bronchiolitis and examining the charts of patients meeting age criteria to determine if they are eligible. They will then walk to the bedside of each eligible infant with bronchiolitis to confirm that supplemental oxygen and room air flow are off and determine the current continuous monitoring status (SpO_2, electrocardiographic, neither, both). Inspired by ecological momentary assessment,[27] this observational approach is the most feasible method to determine true continuous monitoring status across a wide range of hospitals with varying chart documentation and physiologic monitor data infrastructures. These in person observations are necessary even if monitor orders exist because the presence of orders for continuous SpO_2 monitoring are poorly predictive of actual monitoring status (data from current 56-hospital study led by same team conducting this study). Sites will transmit data using REDCap, a secure research data management tool.[28] At the conclusion of the intervention period, questionnaires will be electronically distributed to hospital staff on each participating unit.

5.2 Chart review

5.2.1 Site investigators will provide the following contextual information about audit and feedback and educational outreach delivery events, using a framework previously described:[29]

- Date of event
- What type of session was held? (audit data review, educational outreach session, email, webinar, etc)
- Who was the audience (role composition, (e.g. nurses and residents from the 3 East unit)
- What information was delivered?
- Were specific behavior changes in response to the data discussed?
- Were structural changes discussed?
- Were other changes discussed?
- Were comparison data discussed?
    - The same hospital's past season performance
    - Other hospitals' past season performance
    - Other hospitals' current season performance
    - Performance of other units in the same hospital during current season
    - Performance of other units in other hospitals during current season
- Were graphs presented?
- How fresh were the most recent data discussed? (e.g. from the previous week)
- Implementation dates and details of any active quality improvement projects occurring concurrently

5.2.2 The following data elements will be abstracted from the chart and stored in the study database:

- Hospital
- Admission date and time
- Coded patient-admission ID – this is an identifier for each unique patient-admission to allow the data analysis to account for clustering of observations within individual patient-admissions. This is not an actual patient identifier assigned by the hospital; it is a study-specific coded identifier with the linkage master list document held by the individual site (no

actual patient identifiers other than the dates listed here are transmitted to CHOP from other sites).

- Unit name
- Bed number
- Patient age
- Patient sex
- Patient race and ethnicity
- Patient primary diagnosis and chronic conditions
- Forms of chronic neurologic impairment (e.g. static encephalopathy, cerebral palsy, hydrocephalus, spina bifida, epilepsy/seizure disorder, hypotonia, other form of neurologic impairment)
- Technology dependence (e.g. short-term feeding tube, long-term feeding tube, central venous line, renal dialysis)
- History of apnea or cyanosis during this illness
- History of ICU stay earlier in this admission
- Patient gestational age
- How long have they been off respiratory support (e.g. supplemental oxygen or room air flow), according to the chart?
- Are any pulse oximetry measurements documented in the chart since the patient has been off respiratory support (e.g. supplemental oxygen or room air flow)?
- Do they have an active continuous pulse oximetry monitoring order in the chart right now?
- Do they have an active continuous electrocardiographic/cardiorespiratory monitoring order in the chart right now?
- For hospitals with integration of medical device data into electronic health records:
  - Is there at least 1 pulse oximetry data point visible in the chart from the same minute that rounds occurred?
  - Is there at least 1 pulse oximetry data point visible in the chart for each of the 15 minutes preceding when rounds occurred?
  - Is there at least 1 pulse oximetry data point visible in the chart for each of the 15 minutes following when rounds occurred?

5.2.3 The following data elements will be abstracted from the chart and stored in the individual site's separate master list alongside the coded ID, admission date, and data collection rounds date but NOT entered into the study database and NOT transmitted outside of the institution where the patient is hospitalized:

- Patient first name
- Patient last name
- Patient medical record number
- Patient admission /account number (a hospital-specific identifier in the EHR that identifies the specific admission)

5.3 Bedside visual observation "rounds"

- Date and time rounds occurred at this patient's bedside (the exact time, to the minute, using the time displayed on the bedside monitor as the time of record)
- Based on observing the patient at the bedside, are they currently receiving any respiratory support (e.g. supplemental oxygen or nasal cannula flow)?
- Are they being continuously pulse oximetry monitored right now based on bedside observation?
- Are they being continuously electrocardiographically/cardiorespiratory monitored (using chest leads to measure heart rate, heart rhythm, and/or respiratory rate) right now based on bedside observation?
- Field notes based on brief real time inquiry of health care professionals, if performed, regarding why the patient is continuously monitored

5.4 Staff questionnaires (see appendix for instrument)

5.4.1 Site PIs will submit to CHOP for recruitment

- Email addresses of potential health professional subjects

5.4.2 The questionnaires will include the following elements:

- Hospital
- Unit(s) where person worked during the intervention period
- Profession (nurse, attending physician, etc.)
- Did the person care for any patients with bronchiolitis on an intervention unit during the intervention period?
- Gender, race, ethnicity of subject completing the questionnaire
- Acceptability of Intervention Measure (AIM)
- Intervention Appropriateness Measure (IAM)
- Feasibility of Intervention Measure (FIM)
- Supplemental items (see appendix)

Please note that we may make minor changes to the items included in the questionnaire in the months between initial IRB submission and questionnaire administration based on ongoing discussion with the investigative team and site PIs. As long as the changes do not represent major changes in scope, we will not submit revised versions of the questionnaire to the IRB.

6 STATISTICAL CONSIDERATIONS

This is a feasibility study preparatory to a clinical trial. Therefore, there is minimal emphasis on statistical power.

6.1 Primary Endpoint

The primary endpoints are the resulting scores on the questionnaire measures above.

6.2 Secondary Endpoints

Secondary endpoints will include the following:

- Continuous pulse oximetry use rates in children with bronchiolitis who are not requiring supplemental oxygen administration.
- The test characteristics (sensitivity, specificity, positive predictive value, negative predictive value) of the electronic health record data from the medical monitoring device for actual monitoring at the bedside in bronchiolitis

6.3 Statistical Methods

6.3.1 Questionnaire-based methods (AIM, IAM, FIM)

We will generate average scores for each item and for each measure, stratified by hospital and role (e.g. nurse, physician).

6.3.2 Pulse oximetry use rates

We will calculate the overall rate of SpO_2 monitoring overuse within each hospital. The *rate numerator* is the number of patients with bronchiolitis who are breathing room air (not requiring any supplemental oxygen) and are continuously SpO_2-monitored. The *rate denominator* is the total number of patients with bronchiolitis who are breathing room air. We will stratify these rates by key variables (e.g. time of day, amount of time off supplemental oxygen).

6.3.3 Medical device integration test characteristics

We will calculate the sensitivity, specificity, positive predictive value, negative predictive value of the electronic health record data from the medical monitoring device for actual monitoring at the bedside in bronchiolitis.

6.4 Sample Size and Power

By engaging 10 sites in a range of different settings, we expect to gain meaningful insight into the feasibility of performing a larger trial of this intervention. By collecting a total of approximately 100 questionnaires on each of the AIM, FIM, and IAM per hospital, we will have 90% power to reject the null hypothesis response of “Neither agree nor disagree” (3.0) assuming a mean response of “Agree” (4.0), alpha=.05, standard deviation=3. A total of 73 responses would provide 80% power to detect the above conditions.

7 SAFETY MANAGEMENT

7.1 Clinical Adverse Events

Clinical adverse events (AEs) will be monitored throughout the study. See details in section 8.4.1 describing the proactive adverse event surveillance plan.

7.2 Adverse Event Reporting

Since the study procedures are not greater than minimal risk, SAEs are not expected. If any unanticipated problems related to the research involving risks to subjects or others happen during the course of this study (including SAEs), they will be reported to the IRB in accordance with CHOP IRB SOP 408: Unanticipated Problems Involving Risks to Subjects. AEs that are not serious but that are notable and could involve risks to subjects will be summarized in narrative or other format and submitted to the IRB at the time of continuing review.

8 STUDY ADMINISTRATION

8.1 Treatment Assignment Methods

This is a single arm study without randomization or blinding.

8.2 Data Collection and Management

8.2.1 Data sources

8.2.1.1 Chart review

Data from chart review will be extracted from the medical record system used at the hospital site. This may include electronic medical records (e.g. the Epic system at CHOP) or paper medical records.

8.2.1.2 Bedside visual observation "rounds"

Data will be obtained by visual observing and documenting the current state of respiratory support and physiologic monitoring occurring in patient rooms.

8.2.1.3 Staff questionnaires

- As listed in Sections 5 and 6

8.3 Confidentiality

All data and records generated during this study will be kept confidential in accordance with Institutional policies and HIPAA on subject privacy. The Investigator and other site personnel will not use such data and records for any purpose other than conducting the study.

The only PHI elements that documented in the study database are the admission date and the date the data collection rounds occurred (which by definition is a date during the patient's hospitalization). Thus this is a *limited dataset*.

The dataset is also *coded* to decrease the risk of breach of confidentiality. A patient-admission ID number not otherwise associated with the individual (not derived from any element of PHI) will be generated by each site and included in the study database allowing patients to be followed over time within the same admission. This is an identifier for each unique patient-admission to allow the data analysis to account for clustering of observations within individual patient-admissions. This is not an actual patient identifier assigned by the hospital; it is a study-specific coded identifier with the linkage master list document held by the individual site (no PHI other than the dates listed above are transmitted to CHOP from other sites).

Hospitals that choose to maintain paper master lists will keep them in locked file cabinets. Hospitals that choose to maintain electronic master lists will store them either on hard drives owned by the institution or servers maintained/approved by their institution. The master list containing the key to decipher the coded dataset will be deleted/destroyed at the earliest possible date that complies with individual hospitals' retention of records policies.

Questionnaire data will not contain any PHI.

No identifiable data will be used for future study without first obtaining IRB approval. The investigator will obtain a data use agreement between provider (the PI) and any recipient researchers (including others at CHOP) before sharing a limited dataset (dates and zip codes).

8.4 Regulatory and Ethical Considerations

8.4.1 Data and Safety Monitoring Plan

The risks of this study are minimal as it involves an intervention intended to align clinical practice with established guidelines and recommendations for quality bronchiolitis care. The Principal Investigator is responsible for data and safety monitoring. The primary risk is breach of privacy and confidentiality. We will also perform proactive surveillance for threats to patient safety.

8.4.1.1 Plan for mitigating and monitoring risk of privacy and confidentiality breaches

CHOP's instance of REDCap will be used for all data collection and management in this study, including data collection performed at non-CHOP sites. For feasibility reasons, study sites will be permitted to use a temporary paper data collection worksheet as long as it is stored in a locked file

cabinet and destroyed after abstraction into REDCap. Handling of the master list is described in the above section.

After data collection, exported data for analysis will take place only on computers that meet CHOP IT standards for encryption and password protection as outlined in CHOP IT SOP A-3-6. Data will be stored on the CHOP research SAN and/or other secure servers maintained by CHOP IT. Only investigators and study staff approved by the IRB will have access to data containing PHI.

In accordance with the current CHOP Retention of Records Policy A-3-9 in the Administrative Policy Manual, section on minimal risk human subjects research records, we will retain all information from this study for 6 years after the study is completed, after which PHI will be stripped from the data set.

We recognize that other sites relying on the CHOP IRB have different record retention requirements. For this reason, we will:

1. Provide all data submitted to CHOP back to the Site PI at the conclusion of the study using a CHOP IT approved secure method, allowing each site to meet local record retention requirements. We will not submit Site-Specific Amendments describing each site's record retention requirement.
2. Support other site-specific record retention or local IRB policy exceptions (such as a local requirement to retain data collection worksheets after the data are abstracted into REDCap) that may differ from what we have written above as long as they are described in the Site Survey or a Site-Specific Amendment.

The CHOP PI will monitor and review the study progress, the accuracy and security of the data, and will ensure subject confidentiality and safety.

8.4.1.2 Plan for proactive surveillance for threats to patient safety

In this study, we will regularly evaluate the safety of the intervention, since the intervention may lead to less intensive monitoring for some bronchiolitis patients. In order to monitor for threats to safety, we will:

- Monitor for code blue team activations and rapid response team / medical emergency team / critical assessment team activations in all bronchiolitis patients hospitalized on units participating in the study during the intervention period. This includes patients who were not subjects of data collection, because the intervention is applied at the unit level and could impact the care of patients with bronchiolitis even if they are not reviewed during data collection rounds.
- Investigators will review the chart of each patient meeting the above criteria to determine if the patient was unmonitored at the time of the event that led to the activation.
- Events meeting the activation criteria in which the patient was unmonitored at the time of the event that led to the activation will be considered study adverse events, whether or not they are related to the research intervention, and be documented in research records as meeting those criteria.
- Investigators will then determine, through chart review and speaking with staff present at the time of the event, if available, if the event was at least possibly related to the study intervention, defined as "there is a reasonable possibility that the adverse event may have been caused by the procedures involved in the research."
- Investigators will follow the CHOP IRB's Unanticipated Problems Decision Tree (https://irb.research.chop.edu/unanticipated-problems) in order to determine whether the Adverse Event requires prompt reporting to the IRB or should be filed with the next continuing review.
- Adverse Events that meet the definition of Unanticipated Problem Involving Risks to Subjects will be reported promptly to the IRB and the study will be suspended pending review of the event by the IRB. Following the review, we will work with the IRB and staff from the inpatient units to determine if the study should be modified to improve the safety of patients.

- Sites other than CHOP will follow their IRB's internal reporting requirements, if they differ from the procedures outlined above.

8.4.2 Risk Assessment

Risks are not greater than minimal in this study. The primary risk is breach of privacy and confidentiality. Less likely are threats to patient safety. These risks will be minimized as described above.

Given that there are national recommendations from Choosing Wisely and the AAP that support using less continuous monitoring in stable bronchiolitis, this study is not introducing any additional risk than would be introduced if clinical leaders initiated efforts into routine clinical care to align their hospitals' practice with the existing national guidelines. The intervention simply aims to measure the feasibility of revealing how hospitals are doing with respect to these guidelines by showing them their own data, and providing information/education on the existing recommendations. All decisions about changes to monitoring are made by the primary medical team, not the researchers.

8.4.3 Potential Benefits of Trial Participation

8.4.3.1 Direct

Patients, their families, and the health professionals caring for them have the potential to benefit from higher quality, guideline-concordant health care. This may produce fewer monitor alarms, and thereby fewer interruptions from alarms that may break the concentration of health professionals and contribute to errors, wake patients from sleep, or cause unnecessary anxiety in patients and their loved ones. The patients also may have a lower risk of harm due to a reduction in the probability of the clinicians caring for them experiencing the alarm fatigue that can result from high alarm rates. Patients may also benefit by not having their hospital stays prolonged unnecessarily due to the overdiagnosis of inconsequential mild hypoxemia.

8.4.3.2 Indirect

The study outlined in this proposal will generate important knowledge that will advance (a) the science of audit and feedback and (b) plans for a future large-scale cluster-randomized trial of this intervention.

8.4.4 Risk-Benefit Assessment

Based on the discussion above and the lack of deviation from standard clinical care, the benefits of this study far outweigh the minimal risks. Given that the risks of the study are minimal, it is reasonable to proceed with the project.

8.5 Recruitment Strategy

The pragmatic approach to recruitment of health care professionals and patients for the intervention has been described in previous sections.

For the data collection in-person observational rounds, site investigators will identify all patients currently admitted to their hospital's non-ICU pediatric wards with bronchiolitis by reviewing the census of each unit that cares for children with bronchiolitis and examining the charts of patients meeting age criteria to determine if they are eligible. Eligibility screening does not involve recording any data until the patient is determined to be eligible and is enrolled.

For the primary objective, measuring feasibility, acceptability, and appropriateness using the validated questionnaire instruments, site PIs will distribute the questionnaire to the following health care professional types who worked on an intervention unit in a role involving the care of patients with bronchiolitis during the intervention period:

- Attending physicians
- Resident physicians

- Nurses
- Nursing assistants
- Physician assistants
- Respiratory therapists
- Other relevant staff (e.g. if a hospital's bronchiolitis care team includes staff who educate parents about their child's condition prior to discharge, we would include them here)

Questionnaires will be distributed in paper and electronic formats. Paper questionnaires will be distributed during unit-based events such as safety huddles,[24,25] staff meetings, and rounds and subsequently entered into a CHOP-based REDCap form by the site investigative team or scanned and emailed to the CHOP-based study email address. Electronic questionnaires will be distributed using CHOP-based REDCap survey distribution tools to track responses and nonresponses. In order to distribute, site PIs will work with unit leadership within each discipline listed above to identify potential eligible health professional subjects. Site PIs will submit a list of email addresses of these potential subjects to CHOP study leadership, who will enter these into the CHOP-based REDCap survey distribution system.

8.6 Informed Consent/Assent and HIPAA Authorization

8.6.1 Informed consent and HIPAA for the primary measure (questionnaires)

Consent will be obtained for the questionnaires administered to health care professional; we request waiver of documentation. The waiver is appropriate under §46.117(c)(ii): the research presents no more than minimal risk of harm to subjects and involves no procedures for which written consent is normally required outside of the research context.

The first section of the questionnaire will include the required elements of consent and a statement that completion of the questionnaire indicates willingness and consent to participate. Specific language is included in the Appendix. HIPAA Authorization is not necessary for the questionnaire portion of the study; email addresses of health care professionals are being collected in order to distribute but are not considered PHI because they do not relate to the health of an individual.

8.6.2 Waiver of Consent and Assent for all other aspects of the study

Waivers of consent and assent are being requested for this study, with the exception of the questionnaire portion described in the section above. If consent was obtained, the only central record directly linking the subject and the research data would be the informed consent form and the principal risk would be potential harm resulting from a breach of confidentiality. In addition, this project involves no procedures for which written consent is normally required outside of the research context. Any health care professional can opt out of study participation at any time simply by not participating in study-related activities.

The research meets the criteria of 45 CFR 46.116(d), as illustrated below:

(1) Risks are not greater than minimal in this study. The primary risk is breach of privacy and confidentiality. The risk will be minimized by keeping all data and records confidential in accordance with institutional policies on subject privacy. The investigators will not use data or records for any purpose other than conducting the study. Please see Section 8.3 for additional details.

(2) The waiver or alteration will not adversely affect the rights and welfare of the subjects because PHI will only be accessed by IRB approved study staff performing data collection, and we are collecting a limited, coded dataset to minimize risk of PHI exposure.

(3) The research could not practicably be carried out without the waiver or alteration. The intent is to obtain a large, unbiased sample of patients with bronchiolitis to obtain insights on hospital-level practices and systems of care related to pulse oximetry monitoring in bronchiolitis. The research could not practicably be carried out without the waiver or alteration, as it may not be feasible to

locate and contact the families of all of the patients whose clinical records will be required in order for this to be an unbiased sample representative of the population with adequate sample size. If we fail to obtain all of the data due to families who cannot be located/contacted, this will threaten the statistical outcome, bias the sample, prohibit conclusions to be drawn or alter the sample such that conclusions would be skewed.

(4) There will not be any pertinent patient-level information that would benefit the patient directly after participation so none of the subjects will be provided with additional information after participation.

8.6.3 Waiver of Assent

Please refer to the justification for waiver of consent above.

8.6.4 Waiver of HIPAA Authorization

We request a waiver of HIPAA Authorization. The study presents no more than minimal risk to the privacy of individuals. The research meets the criteria of 45 CFR 46.116(d), as illustrated below:

(A) The use or disclosure of protected health information involves no more than a minimal risk to the privacy of individuals, based on, at least, the presence of the following elements:

(1) An adequate plan to protect the identifiers from improper use and disclosure;

- *We have provided an adequate plan to protect identifiers from improper use and disclosure above.*

(2) An adequate plan to destroy the identifiers at the earliest opportunity consistent with conduct of the research, unless there is a health or research justification for retaining the identifiers or such retention is otherwise required by law; and

- *We have provided a plan to destroy the identifiers, including those in the master list, at the earliest opportunity consistent with the conduct of the research that is also consistent with institutional record retention policies, described above.*

(3) Adequate written assurances that the protected health information will not be reused or disclosed to any other person or entity, except as required by law, for authorized oversight of the research project, or for other research for which the use or disclosure of protected health information would be permitted by this subpart;

- *Protected health information will not be reused or disclosed to any other entity except as required by law, for authorized oversight of the research project, or for other research for which the use or disclosure of protected health information would be permitted by this subpart.*

(B) The research could not practicably be conducted without the waiver or alteration; and

- *The research could not practicably be conducted without the waiver as described above in waiver of consent request.*

(C) The research could not practicably be conducted without access to and use of the protected health information.

- *The research could not practicably be conducted without access to and use of protected health information needed to track patients over time and to track the dates of admission and data collection at each site during the bronchiolitis season.*

8.7 Payment to Subjects/Families

None.

9 PUBLICATION

Peer-reviewed publication is planned. No identifiable information will be used in publication.

10 APPENDIX

10.1 Consent language for questionnaire

See eIRB application section 12.01 3.0.

10.2 Draft questionnaire items

See eIRB application section 12.02 2.0.

11 REFERENCES

1. Cullen DJ, Nemeskal AR, Cooper JB, Zaslavsky A, Dwyer MJ. Effect of pulse oximetry, age, and ASA physical status on the frequency of patients admitted unexpectedly to a postoperative intensive care unit and the severity of their anesthesia-related complications. *Anesth Analg*. 1992;74(2):181-188.

2. Ochroch EA, Russell MW, Hanson WC, et al. The impact of continuous pulse oximetry monitoring on intensive care unit admissions from a postsurgical care floor. *Anesth Analg*. 2006;102(3):868-875. doi:10.1213/01.ane.0000195583.76486.c4

3. McCulloh R, Koster M, Ralston S, et al. Use of intermittent vs continuous pulse oximetry for nonhypoxemic infants and young children hospitalized for bronchiolitis: a randomized clinical trial. *JAMA Pediatr*. 2015;169(10):898-904. doi:10.1001/jamapediatrics.2015.1746

4. Schuh S, Freedman S, Coates A, et al. Effect of oximetry on hospitalization in bronchiolitis: a randomized clinical trial. *JAMA*. 2014;312(7):712-718. doi:10.1001/jama.2014.8637

5. Cunningham S, Rodriguez A, Adams T, et al. Oxygen saturation targets in infants with bronchiolitis (BIDS): a double-blind, randomised, equivalence trial. *Lancet*. 2015;386(9998):1041. doi:10.1016/S0140-6736(15)00163-4

6. Principi T, Coates AL, Parkin PC, Stephens D, DaSilva Z, Schuh S. Effect of oxygen desaturations on subsequent medical visits in infants discharged from the emergency department with bronchiolitis. *JAMA Pediatr*. 2016;170(6):602-608. doi:10.1001/jamapediatrics.2016.0114

7. Schondelmeyer AC, Simmons JM, Statile AM, et al. Using quality improvement to reduce continuous pulse oximetry use in children with wheezing. *Pediatrics*. 2015;135(4):e1044-e1051. doi:10.1542/peds.2014-2295

8. Schroeder AR, Marmor AK, Pantell RH, Newman TB. Impact of pulse oximetry and oxygen therapy on length of stay in bronchiolitis hospitalizations. *Arch Pediatr Adolesc Med*. 2004;158(6):527. doi:10.1001/archpedi.158.6.527

9. Cunningham S, McMurray A. Observational study of two oxygen saturation targets for discharge in bronchiolitis. *Arch Dis Child*. 2012;97(4):361-363. doi:10.1136/adc.2010.205211

10. McBride SC, Chiang VW, Goldmann DA, Landrigan CP. Preventable adverse events in infants hospitalized with bronchiolitis. *Pediatrics*. 2005;116(3):603-608. doi:10.1542/peds.2004-2387

11. Bonafide CP, Lin R, Zander M, et al. Association between exposure to nonactionable physiologic monitor alarms and response time in a children's hospital. *J Hosp Med*. 2015;10(6):345-351.

12. Bonafide CP, Localio AR, Holmes JH, et al. Video analysis of factors associated with response time to physiologic monitor alarms in a children's hospital. *JAMA Pediatr*. 2017;171(6):524-531.

13. Ralston SL, Lieberthal AS, Meissner HC, et al. Clinical practice guideline: the diagnosis, management, and prevention of bronchiolitis. *Pediatrics*. 2014;134(5):e1474-e1502.

14. Quinonez RA, Garber MD, Schroeder AR, et al. Choosing wisely in pediatric hospital medicine: Five opportunities for improved healthcare value. *J Hosp Med*. 2013;8(9):479-485.

15. Quinonez RA, Coon ER, Schroeder AR, Moyer VA. When technology creates uncertainty: pulse oximetry and overdiagnosis of hypoxaemia in bronchiolitis. *BMJ*. 2017;358:j3850. doi:10.1136/bmj.j3850

16. Cunningham S, Rodriguez A, Boyd KA, McIntosh E, Lewis SC, on behalf of the BIDS Collaborators Group. Bronchiolitis of Infancy Discharge Study (BIDS): a multicentre, parallel-group, double-blind, randomised controlled, equivalence trial with economic evaluation. *Health Technol Assess*. 2015;19(71):1-172. doi:10.3310/hta19710

17. Coon ER, Quinonez RA, Moyer VA, Schroeder AR. Overdiagnosis: how our compulsion for diagnosis may be harming children. *Pediatrics*. 2014;134(5):1013-1023.

18. Monitoring OXYgen in Infants Hospitalized With Bronchiolitis: A Best Practices Trial (The MOXY Trial) - ClinicalTrials.gov. https://clinicaltrials.gov/ct2/show/NCT01646606. Accessed September 15, 2017.

19. Oxygen Saturation Monitoring in Bronchiolitis - ClinicalTrials.gov. https://clinicaltrials.gov/ct2/show/NCT02947204. Accessed September 15, 2017.

20. Sanjay Mahant, MD MSc, FRCPC. Bronchiolitis RCTs at The Hospital for Sick Children, Toronto. September 2017.

21. Rasooly IR, Beidas RS, Wolk CB, et al. Measuring overuse of continuous pulse oximetry in bronchiolitis and developing strategies for large-scale deimplementation: study protocol for a feasibility trial. *Pilot Feasibility Stud*. 2019;5(1):68. doi:10.1186/s40814-019-0453-2

22. Chan WV, Pearson TA, Bennett GC, et al. ACC/AHA special report: Clinical practice guideline implementation strategies: A summary of systematic reviews by the NHLBI Implementation Science Work Group: A report of the American College of Cardiology/American Heart Association Task Force on clinical practice guidelines. *Circulation*. 2017;135(9):e122-e137.

23. Loudon K, Treweek S, Sullivan F, Donnan P, Thorpe KE, Zwarenstein M. The PRECIS-2 tool: designing trials that are fit for purpose. *BMJ*. 2015;350:h2147. doi:10.1136/bmj.h2147

24. Bonafide CP, Localio AR, Stemler S, et al. Safety huddle intervention for reducing physiologic monitor alarms: A hybrid effectiveness-implementation cluster randomized trial. *J Hosp Med*. February 2018:[Epub ahead of print].

25. Dewan M, Wolfe H, Lin R, et al. Impact of a safety huddle–based intervention on monitor alarm rates in low-acuity pediatric intensive care unit patients. *J Hosp Med*. 2017;12(8):652-657.

26. Weiner BJ, Lewis CC, Stanick C, et al. Psychometric assessment of three newly developed implementation outcome measures. *Implement Sci*. 2017;12(1):108. doi:10.1186/s13012-017-0635-3

27. Shiffman S, Stone AA, Hufford MR. Ecological momentary assessment. *Annu Rev Clin Psychol*. 2008;4:1-32.

28. Harris PA, Taylor R, Thielke R, Payne J, Gonzalez N, Conde JG. Research electronic data capture (REDCap)—a metadata-driven methodology and workflow process for providing translational research informatics support. *J Biomed Inf*. 2009;42:377-381.

29. Colquhoun H, Michie S, Sales A, et al. Reporting and design elements of audit and feedback interventions: a secondary review. *BMJ Qual Saf*. 2017;26(1):54-60. doi:10.1136/bmjqs-2015-005004
