# Supplementary material for: Evaluation of an Educational Outreach and Audit and Feedback Program to Reduce Continuous Pulse Oximetry Use in Hospitalized Infants With Stable Bronchiolitis: A Nonrandomized Clinical Trial
Source: JAMA Netw Open. 2021 Sep 2;4(9):e2122826. doi: 10.1001/jamanetworkopen.2021.22826 (PMC8414187; doi:10.1001/jamanetworkopen.2021.22826)
Supplement: Supplement 2. — eTable 1. Questionnaire Response Rates by Site and Role eTable 2. Clinician Questionnaire Responses eFigure 1. Sample Clinician Educational Material eFigure 2. Sample Feedback Dashboard eAppendix. Questionnaire [file jamanetwopen-e2122826-s002.pdf]

Supplementary Online Content

Schondelmeyer AC, Bettencourt AP, Xiao R, et al. Evaluation of an educational outreach and audit and feedback program to reduce continuous pulse oximetry use in hospitalized infants with stable bronchiolitis: a nonrandomized clinical trial. *JAMA Netw Open.* 2021;4(9):e2122826. doi:10.1001/jamanetworkopen.2021.22826

eTable 1. Questionnaire Response Rates by Site and Role
eTable 2. Clinician Questionnaire Responses
eFigure 1. Sample Clinician Educational Material
eFigure 2. Sample Feedback Dashboard
eAppendix. Questionnaire

This supplementary material has been provided by the authors to give readers additional information about their work.

© 2021 Schondelmeyer AC et al. *JAMA Network Open.*

eTable 1. Questionnaire Response Rates by Site and Role

	**Response rate, No. (%)**
Hospital A	156 / 237 (66)
Hospital B	20 / 25 (80)
Hospital C	153 / 207 (74)
Hospital D	131 / 196 (67)
Hospital E	177 / 210 (84)
Hospital F	210 / 318 (66)
Overall	847 / 1193 (71)
By Role	
Attending Physician	177 / 198 (89)
Resident Physician	223 / 325 (72)
Fellow Physician	4 / 4 (100)
Nurse	426 / 658 (65)
Advanced Practice Provider	7 / 8 (88)

© 2021 Schondelmeyer AC et al. *JAMA Network Open.*

eTable 2. Clinician Questionnaire Responses

	Physician No., %	Nurse No., %	Total No., %
**Audit and Feedback Acceptability**			
I like the data feedback. (P=0.01)			
Completely Disagree	1/293 (0)	0/366 (0)	1/659 (0)
Disagree	1/293 (0)	1/366 (0)	2/659 (0)
Neither Agree nor Disagree	17/293 (6)	29/366 (8)	46/659 (7)
Agree	117/293 (40)	188/366 (51)	305/659 (46)
Completely Agree	157/293 (54)	148/366 (40)	305/659 (46)
I welcome continued data feedback about our use of $cSpO_2$ in bronchiolitis. (P=0.00)			
Completely Disagree	1/293 (0)	0/366 (0)	1/659 (0)
Disagree	2/293 (1)	1/366 (0)	3/659 (3)
Neither Agree nor Disagree	10/293 (3)	14/366 (4)	24/659 (4)
Agree	104/293 (36)	186/366 (51)	260/659 (44)
Completely Agree	176/293 (60)	165/366 (45)	341/659 (52)
**Audit and Feedback Feasibility**			
Data feedback about our use of continuous pulse oximetry in bronchiolitis is easy to implement. (P=0.00)			
Completely Disagree			
Disagree	0/293 (0)	0/366 (0)	0/659 (0)
Neither Agree nor Disagree	10/293 (3)	10/366 (3)	20/659 (3)
Agree	52/293 (18)	34/366 (9)	86/659 (13)
Completely Agree	128/293 (44)	203/366 (55)	331/659 (50)
	103/293 (35)	119/366 (33)	222/659 (34)
**Audit and Feedback Appropriateness**			
Data feedback about our use of continuous pulse oximetry in bronchiolitis seems like a good match for our non-ICU floors that care for bronchiolitis. (P=0.11)			
Completely Disagree	1/293 (0)	0/366 (0)	1/659 (0)
Disagree	1/293 (0)	3/366 (1)	4/659 (1)
Neither Agree nor Disagree	11/293 (4)	26/366 (7)	37/659 (6)
Agree	144/293 (49)	193/366 (53)	337/659 (51)
Completely Agree	136/293 (46)	144/366 (39)	280/659 (43)
**Education Acceptability**			
I like the educational sessions. (P=0.28)			
Completely Disagree	0/193 (0)	0/275 (0)	0/468 (0)
Disagree	0/193 (0)	0/275 (0)	0/468 (0)
Neither Agree nor Disagree	18/193 (9)	20/275 (7)	38/468 (8)
Agree	97/193 (49)	154/275 (56)	248/468 (53)
Completely Agree	81/193 (42)	101/275 (37)	182/468 (39)
I welcome continued educational sessions about appropriate use of $cSpO_2$ in bronchiolitis. (P=0.44)			
Completely Disagree	0/193 (0)	0/275 (0)	0/468 (0)
Disagree	3/193 (2)	1/275 (0)	4/468 (1)
Neither Agree nor Disagree	7/193 (4)	8/275 (3)	15/468 (3)
Agree	78/193 (40)	123/275 (45)	201/468 (43)
Completely Agree	105/193 (54)	143/275 (52)	248/468 (53)
**Education Feasibility**			
Education sessions about the use of continuous pulse oximetry in bronchiolitis are easy to implement on our non-ICU floors that care for bronchiolitis. (P=0.52)			
Completely Disagree	0/193 (0)	0/275 (0)	0/468 (0)
Disagree	2/193 (1)	4/275 (1)	6/468 (1)
Neither Agree nor Disagree	22/193 (11)	21/275 (8)	43/468 (9)
Agree	103/193 (53)	158/275 (57)	261/468 (56)
Completely Agree	66/193 (34)	92/275 (33)	158/468 (34)
**Education Appropriateness**			
Educational sessions about the appropriate use of continuous pulse oximetry in bronchiolitis seem like a good match for our non-ICU floors that care for bronchiolitis. (P=0.21)			

© 2021 Schondelmeyer AC et al. *JAMA Network Open.*

	Physician No., %	Nurse No., %	Total No., %
Completely Disagree	0/193 (0)	0/275 (0)	0/468 (0)
Disagree	0/193 (0)	0/275 (0)	0/468 (0)
Neither Agree nor Disagree	6/193 (3)	10/275 (4)	16/468 (3)
Agree	84/193 (44)	141/275 (51)	225/468 (48)
Completely Agree	103/193 (53)	124/275 (45)	227/468 (49)
**Deimplementation Intervention Safety**			
Intermittently spot-checking oxygen saturation instead of cSpO₂ in stable, uncomplicated patients is safe. (P=0.00)			
Completely Disagree	0/414 (0)	0/426 (0)	0/840 (0)
Disagree	1/414 (0)	5/426 (1)	6/840 (1)
Neither Agree nor Disagree	7/414 (2)	30/426 (7)	37/840 (4)
Agree	116/414 (28)	220/426 (52)	336/840 (40)
Completely Agree	290/414 (70)	171/426 (40)	461/840 (55)
Intermittently spot-checking oxygen saturation instead of cSpO₂ in stable, uncomplicated patients is upsetting to parents. (P=0.00)			
Completely Disagree	44/414 (11)	18/426 (4)	62/840 (7)
Disagree	224/414 (54)	177/426 (42)	401/840 (48)
Neither Agree nor Disagree	111/414 (27)	138/426 (32)	249/840 (30)
Agree	30/414 (7)	74/426 (17)	104/840 (12)
Completely Agree	5/414 (1)	19/426 (5)	24/840 (3)
Intermittently spot-checking oxygen saturation instead of cSpO₂ in stable, uncomplicated patients is a good idea. (P=0.00)			
Completely Disagree	0/414 (0)	0/426 (0)	0/840 (0)
Disagree	1/414 (0)	7/426 (2)	8/840 (1)
Neither Agree nor Disagree	9/414 (2)	46/426 (11)	55/840 (7)
Agree	118/414 (29)	205/426 (48)	323/840 (38)
Completely Agree	286/414 (69)	168/426 (39)	454/840 (54)
Intermittently spot-checking oxygen saturation instead of cSpO₂ in stable, uncomplicated patients puts patients at risk. (P=0.00)			
Completely Disagree	130/414 (31)	49/426 (12)	179/840 (21)
Disagree	251/414 (61)	256/426 (61)	507/840 (60)
Neither Agree nor Disagree	23/414 (6)	94/426 (22)	117/840 (14)
Agree	7/414 (2)	22/426 (5)	29/840 (3)
Completely Agree	3/414 (1)	5/426 (1)	8/840 (1)
Intermittently spot-checking instead of cSpO₂ in stable, uncomplicated bronchiolitis patients could help us reduce length of stay. (P=0.00)			
Completely Disagree	2/414 (0)	1/426 (0)	3/840 (0)
Disagree	2/414 (0)	8/426 (2)	10/840 (1)
Neither Agree nor Disagree	9/414 (2)	48/426 (11)	57/840 (7)
Agree	147/414 (36)	228/426 (54)	375/840 (45)
Completely Agree	254/414 (61)	141/426 (33)	395/840 (47)
Intermittent checking instead of cSpO₂ in stable, uncomplicated bronchiolitis patients could help us reduce monitor alarm fatigue. (P=0.07)			
Completely Disagree	3/414 (1)	2/426 (1)	5/840 (1)
Disagree	0/414 (0)	2/426 (1)	2/840 (0)
Neither Agree nor Disagree	5/414 (1)	11/426 (3)	16/840 (2)
Agree	108/414 (26)	137/426 (32)	245/840 (29)
Completely Agree	298/414 (72)	274/426 (64)	572/840 (68)
Most of my [nurse / physician] colleagues prefer intermittently spot-checking oxygen saturation instead of using cSpO₂ in stable, uncomplicated bronchiolitis patients. (P=0.00)			
Completely Disagree	0/414 (0)	3/426 (1)	3/840 (0)
Disagree	13/414 (3)	45/426 (11)	58/840 (7)
Neither Agree nor Disagree	56/414 (14)	101/426 (24)	157/840 (19)
Agree	165/414 (40)	170/426 (40)	335/840 (40)
Completely Agree	180/414 (43)	107/426 (25)	287/840 (34)

© 2021 Schondelmeyer AC et al. *JAMA Network Open.*

	Physician<br>No., %	Nurse<br>No., %	Total<br>No., %
Going forward, I intend to intermittently spot check oxygen saturation instead of using $cSpO_2$ in stable, uncomplicated bronchiolitis patients. (P=0.00)			
Completely Disagree	0/414 (0)	1/426 (0)	1/840 (0)
Disagree	1/414 (0)	2/426 (1)	3/840 (0)
Neither Agree nor Disagree	17/414 (4)	42/426 (10)	59/840 (7)
Agree	135/414 (33)	210/426 (49)	345/840 (41)
Completely Agree	264/414 (63)	171/426 (40)	432/840 (51)

Note: The p-value represents the overall Pearson Chi2 test of independence.

© 2021 Schondelmeyer AC et al. *JAMA Network Open.*

eFigure 1. Sample Clinician Educational Material

emo SpO₂

What do the **national guidelines** say about using continuous pulse oximetry in bronchiolitis?

**Choosing Wisely** (2013)	**Do not** use continuous pulse oximetry routinely in children **unless** they are on supplemental oxygen
**AAP** (2014)	Clinicians may choose not to use continuous pulse oximetry
**BEEP** (2019)	**Transition** children from continuous to intermittent pulse oximetry **within 1 hr of weaning** O_2 to room air if SpO_2 >=90%

What do our **local guidelines** say about using continuous pulse oximetry in bronchiolitis?

When to **definitely** monitor continuously:

- **Severe** bronchiolitis (including, but not limited to use of HFNC)

When to **avoid** continuous monitoring:

- **Mild** or **moderate** bronchiolitis
- Preparing for d/c (our guidelines say nothing about waiting for a nap!)

What are the **downsides** to continuous monitoring in bronchiolitis?

- ↑ Alarm fatigue
- ↑ Length of stay
- ↑ Anxiety about mild desats

What Can I Do To Reduce SpO_2 Overuse?

- **Plan** for transition to intermittent spot checks
- **Check in** with FLOCs about status of transition plan
- **Communicate** early if you have concerns about taking off pulse ox

© 2021 Schondelmeyer AC et al. *JAMA Network Open.*

eFigure 2. Sample Feedback Dashboard

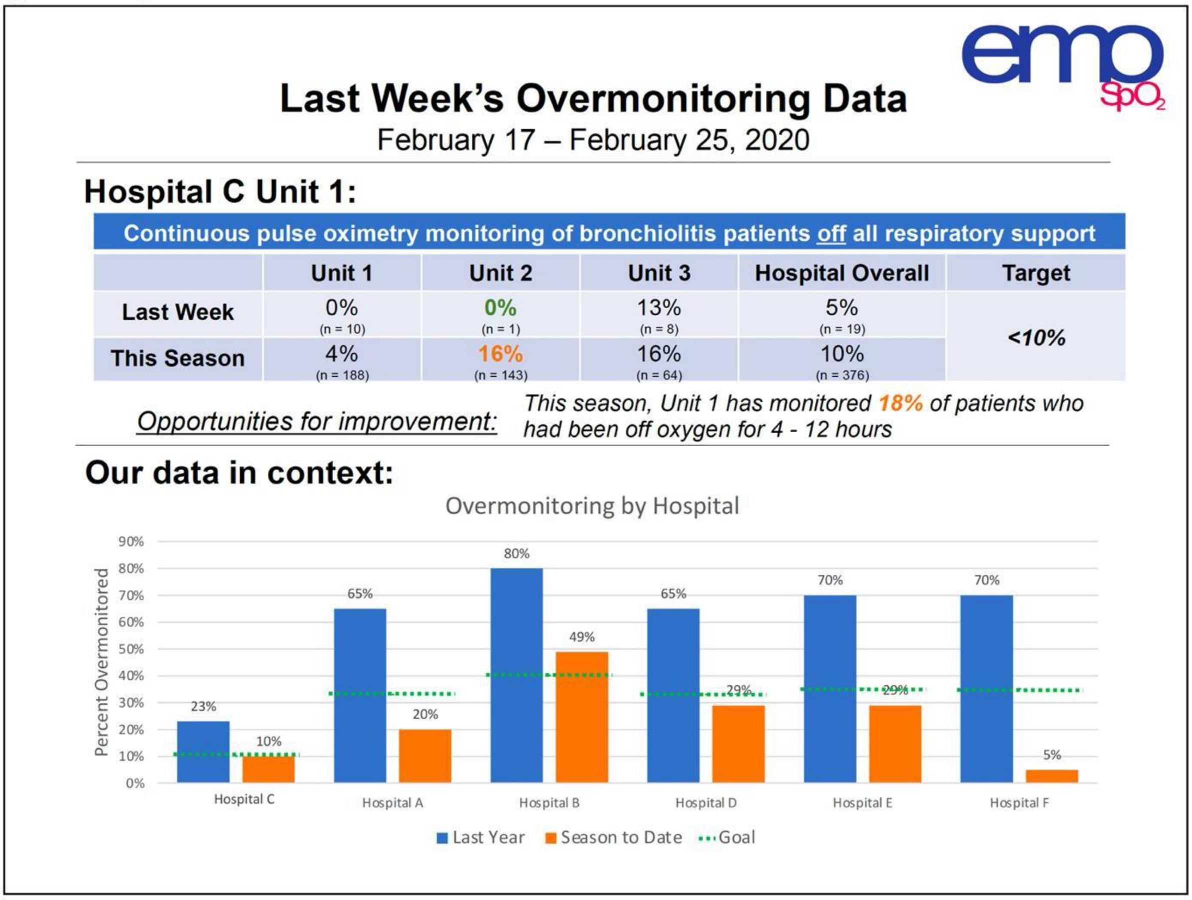

© 2021 Schondelmeyer AC et al. *JAMA Network Open.*

eAppendix. Questionnaire

Please answer the questions below to the best of your ability. Thank you!

In this survey you will find a series of questions about the use of continuous pulse oximetry monitoring in bronchiolitis. We are interested in your responses because, in the past few months, you worked on a unit that was part of a study exploring ways to align continuous pulse oximetry use in stable bronchiolitis with national guideline recommendations. Attached, you will find additional information that may help you decide if you want to take part in the study. Participation is voluntary, and your decision to participate (or not participate) in the research will not affect your performance evaluation or employment. Your response will not be shared with your supervisor. Completion of the questionnaire indicates your willingness to participate and serves as your consent.

Your profession:

- ◯ Attending physician
- ◯ Resident physician
- ◯ Fellow physician
- ◯ Nurse
- ◯ Advanced practice nurse/nurse practitioner
- ◯ Physician assistant
- ◯ Respiratory therapist
- ◯ None of the above

Do you have a leadership role on your Unit or in your Section, Division, or Department?

- ◯ Yes
- ◯ No

We recognize that the current pandemic has probably impacted your practice, and that it would drastically impact the implementation of any new intervention. For the purposes of this survey, please assume that:

- None of the patients have suspected or confirmedCOVID-19.

- Your hospital is normally staffed and not in the midst of apandemic.

The survey questions refer to "STABLE" and "UNCOMPLICATED" bronchiolitis patients:

"STABLE" means they are not presently requiring supplemental oxygen or flow, and their oxygen saturation is 90% or higher.

"UNCOMPLICATED" means they do not have significant comorbidities.

On one or more of the units where you worked this winter, we implemented 2 interventions to try and decrease unnecessary pulse oximetry in bronchiolitis. They were: educational sessions and data feedback.

Between December 2019 and March 2020... did you ever attend any educational sessions about appropriate use of continuous pulse oximetry in stable, uncomplicated bronchiolitis patients?
(These sessions included one or more of the following: Discussing of the guidelines and evidence that recommend avoiding continuous pulse oximetry in stable, uncomplicated bronchiolitis Reviewing your hospital's local guidelines/policies/pathways for using pulse oximetry in bronchiolitis Discussing the natural history of brief, self-limited desaturations in bronchiolitis)

◯ Yes ◯ No ◯ Not sure

Please answer the following questions about the educational sessions discussing appropriate use of continuous pulse oximetry in stable, uncomplicated bronchiolitis on our non-ICU floors that care for bronchiolitis.

	Completely Agree	Agree	Neither agree nor disagree	Disagree	Completely disagree
I like the educational sessions	◯	◯	◯	◯	◯

© 2021 Schondelmeyer AC et al. *JAMA Network Open.*

Educational sessions about appropriate use of continuous pulse oximetry in bronchiolitis are easy to implement on our non-ICU floors that care for bronchiolitis.	○	○	○	○	○
Educational sessions about appropriate use of continuous pulse oximetry in bronchiolitis seem like a good match for our non-ICU floors that care for bronchiolitis.	○	○	○	○	○
I welcome continued educational sessions about appropriate use of continuous pulse oximetry in bronchiolitis.	○	○	○	○	

Between December 2019 and March 2020... were you ever provided with data about your unit/department's use of continuous pulse oximetry in stable, uncomplicated bronchiolitis patients?
(Data feedback includes emails with data and/or sessions involving a physician, nurse, or research staff member discussing your unit/department's performance in reducing pulse oximetry use using data from the prior week in a huddle or other group setting)

○ Yes ○ No ○ Not sure

Please answer the following questions regarding the data feedback about your unit/department's use of continuous pulse oximetry in stable, uncomplicated bronchiolitis.

	Completely agree	Agree	Neither agree nor disagree	Disagree	Completely disagree
I like the data feedback.	○	○	○	○	○
Data feedback about our use of continuous pulse oximetry in bronchiolitis is easy to implement.	○	○	○	○	○
Data feedback about our use of continuous pulse oximetry in bronchiolitis seems like a good match for our non-ICU floors that care for bronchiolitis.	○	○	○	○	○
I welcome continued data feedback about our use of continuous pulse oximetry in bronchiolitis.	○	○	○	○	○

Please answer the following questions about:
intermittently spot-checking the oxygen saturation instead of using continuous pulse oximetry monitoring in stable, uncomplicated bronchiolitis patients.

	Completely agree	Agree	Neither agree nor disagree	Disagree	Completely disagree
Intermittently spot-checking oxygen saturation instead of continuous monitoring is safe	○	○	○	○	○

© 2021 Schondelmeyer AC et al. *JAMA Network Open.*

Intermittently spot-checking oxygen saturation instead of continuous monitoring in stable, uncomplicated bronchiolitis patients is upsetting to parents.	◯	◯	◯	◯	◯
Intermittently spot-checking oxygen saturation instead of continuous monitoring in stable, uncomplicated bronchiolitis patients is a good idea.	◯	◯	◯	◯	◯
Intermittently spot-checking oxygen saturation instead of continuous monitoring instable, uncomplicated bronchiolitis patients puts patients at risk.	◯	◯	◯	◯	◯
Intermittently spot-checking ◯ oxygen saturation instead of continuous monitoring in stable, uncomplicated bronchiolitis patients could help us reduce their hospital length of stay.	◯	◯	◯		○
Intermittently spot-checking ◯ oxygen saturation instead of continuous monitoring in stable, uncomplicated bronchiolitis patients could help us reduce monitor alarm fatigue.	◯	◯	◯		○
Most of my [profession] colleagues prefer intermittently spot checking oxygen saturation instead of using continuous monitoring in stable, uncomplicated bronchiolitis patients.	◯	◯	◯	◯	○
Going forward, I intend to ◯ intermittently spot check oxygen saturation instead of using continuous monitoring instable uncomplicated bronchiolitis patients.	◯	◯	◯		○

Please answer the following optional questions about your demographics, for aggregate reporting purposes only:

Your sex:	◯ Female<br>◯ Male<br>◯ Other<br>◯ Prefer not to say
Ethnicity you most closely identify with:	◯ Hispanic or Latino<br>◯ Not Hispanic or Latino<br>◯ Prefer not to say

© 2021 Schondelmeyer AC et al. *JAMA Network Open.*

Race(s) you most closely identify with (select all Native that apply):

- ☐ American Indian or AlaskaAsian
- ☐ Black or African-American
- ☐ Native Hawaiian or Other Pacific Islander
- ☐ White
- ☐ Other
- ☐ Prefer not to say

© 2021 Schondelmeyer AC et al. *JAMA Network Open.*

A description of this clinical trial will be available on http://www.ClinicalTrials.gov, as required by U.S. law. This website will not include information that can identify you. At most, the website will include a summary of the results. You can search this website at any time.

© 2021 Schondelmeyer AC et al. *JAMA Network Open.*
