# Supplementary material for: Evaluation of an Educational Outreach and Audit and Feedback Program to Reduce Continuous Pulse Oximetry Use in Hospitalized Infants With Stable Bronchiolitis: A Nonrandomized Clinical Trial
Source: JAMA Netw Open. 2021 Sep 2;4(9):e2122826. doi: 10.1001/jamanetworkopen.2021.22826 (PMC8414187; doi:10.1001/jamanetworkopen.2021.22826)
Supplement: Supplement 3. — Nonauthor Collaborators [file jamanetwopen-e2122826-s003.pdf]

Supplemental Online Content: Nonauthor Collaborators

*Indicates required information. Only first name, last name, and suffix will appear in PubMed.

*Group Name(s): Pediatric Research in Inpatient Settings (PRIS) Network							
***First Name and Middle Initial(s)**	***Last Name**	***Suffix (eg, Jr, III)**	Academic Degrees	Institution	Location (city, state/province, country)	Role or Contribution, eg, chair, principal investigator	Group (if more than 1 Group listed in the byline) and/or Subgroup (eg, Steering Committee)
Kyle A	Lamphier		MD	Children's Hospital Los Angeles; Keck School of Medicine, University of Southern California	Los Angeles, CA, USA	Co-investigator	
Maria	Santos		MD	Children's Hospital Los Angeles; (currently at Providence Cedars-Sinai Tarzana Medical Center)	Los Angeles, CA, USA (currently Tarzana, CA, USA)	Co-investigator	
Susan	Wu		MD	Children's Hospital Los Angeles; Keck School of Medicine, University of Southern California	Los Angeles, CA, USA	Co-investigator	
Phillip A	Abarca		now MD, MPH	Keck School of Medicine, University of Southern California (currently at Keck School of Medicine, University of Southern California, Internal Medicine Residency Program)	Los Angeles, CA, USA	Research Assistant	
Amir	Hassan		BA	Children's Hospital Los Angeles (now Columbia University Vagelos College of Physicians and Surgeons)	New York, NY, USA	Research Assistant	
Sabrina	Sedano		BS	Children's Hospital Los Angeles	Los Angeles, CA, USA	Research Assistant	
Jennifer R.	Moore		MD	Seattle Children's Hospital	Seattle, WA, USA	Data collection and provider peer education	
Joshua	Frankland		MD	Seattle Children's Hospital	Seattle, WA, USA	Data collection and provider peer education	

Supplemental Online Content: Nonauthor Collaborators

*Indicates required information. Only first name, last name, and suffix will appear in PubMed.

*First Name and Middle Initial(s)	*Last Name	*Suffix (eg, Jr, III)	Academic Degrees	Institution	Location (city, state/province, country)	Role or Contribution, eg, chair, principal investigator	Group (if more than 1 Group listed in the byline) and/or Subgroup (eg, Steering Committee)
Kristin	Van Genderen		MD	Ann & Robert H. Lurie Children's Hospital of Chicago	Chicago, IL, USA	Data collection, eductational session facilitation	
M. Katherine	Stone		MD, MPH	Ann & Robert H. Lurie Children's Hospital of Chicago	Chicago, IL, USA	Data collection	
Michael	Spewak		MD	Ann & Robert H. Lurie Children's Hospital of Chicago	Chicago, IL, USA	Data collection	
Victoria	Rodriguez		MD	Ann & Robert H. Lurie Children's Hospital of Chicago	Chicago, IL, USA	Data collection	
Waheeda	Samady		MD, MSCI	Ann & Robert H. Lurie Children's Hospital of Chicago	Chicago, IL, USA	Data collection	
Amera	Al-Ali		BSN, RN, CPN	Ann & Robert H. Lurie Children's Hospital of Chicago	Chicago, IL, USA	Educational session facilitation	
Huong	Mai		BSN, RN, CPN	Ann & Robert H. Lurie Children's Hospital of Chicago	Chicago, IL, USA	Educational session facilitation	
Laura	El-Hage		MD	Children's	Philadelphia,PA	Data Collection	
Stan	Oliveira		RN, BSN	Children's Hospital of Philadelphia	Philadelphia,PA	Data Collection, Preparation and presentation of education materials	
Jessica	Hart		MD	Children's Hospital of Philadelphia	Philadelphia,PA	Planning approach to engage support from key stake holders, constant feedback and review of survey and education materials	

Supplemental Online Content: Nonauthor Collaborators

*Indicates required information. Only first name, last name, and suffix will appear in PubMed.

***First Name and Middle Initial(s)**	***Last Name**	***Suffix (eg, Jr, III)**	Academic Degrees	Institution	Location (city, state/province, country)	Role or Contribution, eg, chair, principal investigator	Group (if more than 1 Group listed in the byline) and/or Subgroup (eg, Steering Committee)
Laura	Goldstein		MD	Children's Hospital of Philadelphia	Philadelphia,PA	Data Collection, Preparation and presentation of education materials and weekly data results to involved units	
Muida	Menon		RN	Children's Hospital of Philadelphia	Philadelphia,PA	Data collection, presentation of education materials and weekly data results to involved unis	
