# Supplementary material for: Evaluation of an Educational Outreach and Audit and Feedback Program to Reduce Continuous Pulse Oximetry Use in Hospitalized Infants With Stable Bronchiolitis: A Nonrandomized Clinical Trial
Source: JAMA Netw Open. 2021 Sep 2;4(9):e2122826. doi: 10.1001/jamanetworkopen.2021.22826 (PMC8414187; doi:10.1001/jamanetworkopen.2021.22826)
Supplement: Supplement 4. — Data Sharing Statement [file jamanetwopen-e2122826-s004.pdf]

Data Sharing Statement

Schondelmeyer. Evaluation of an Educational Outreach and Audit and Feedback Program to Reduce Continuous Pulse Oximetry Use in Hospitalized Infants With Stable Bronchiolitis. *JAMA Netw Open*. Published September 02, 2021.
doi:10.1001/jamanetworkopen.2021.22826

Data
Data available: Yes
Data types: Deidentified participant data
How to access data: Once a data sharing agreement is in place, in accordance with the policies determined by the study team, CHOP, PRIS, and NIH/NHLBI, we will provide releasable data to investigators under our own auspices via either a secure file transfer mechanism approved by the Research Information Systems Department at CHOP and the receiving institution. PI Email: bonafide@chop.edu
When available: With publication

Supporting Documents
Document types: None

Additional Information
Who can access the data: We will make the data available to users only under an approved data-sharing agreement.
Types of analyses: (1) a commitment to using the data only for research purposes and not to identify any individual participant; (2) IRB approval; (3) a commitment to securing the data using appropriate computer technology; and (4) a commitment to and an agreed-upon plan for destroying the data after analyses are completed.
Mechanisms of data availability: With a signed data access agreement.
Any additional restrictions: The Final Research Data (the dataset necessary to document and support research findings) will be made available for sharing after the main research findings from the final data set have been accepted for publication in a peer reviewed journal. Prior to sharing, data will redacted to strip all direct identifiers of individuals and hospitals. We will make the data available to users only under a data-sharing agreement that provides for: (1) a commitment to using the data only for research purposes and not to identify any individual participant; (2) IRB approval; (3) a commitment to securing the data using appropriate computer technology; and (4) a commitment to and an agreed-upon plan for destroying the data after analyses are

completed. Once a data sharing agreement is in place, in accordance with the policies determined by the study team, CHOP, PRIS, and NIH/NHLBI, we will provide releasable data to investigators under our own auspices via either a secure file transfer mechanism approved by the Research Information Systems Department at CHOP and the receiving institution.
